# Estimating the contribution of key populations towards the spread of HIV in Dakar, Senegal

**DOI:** 10.1002/jia2.25126

**Published:** 2018-07-22

**Authors:** Christinah Mukandavire, Josephine Walker, Sheree Schwartz, Marie‐Claude Boily, Leon Danon, Carrie Lyons, Daouda Diouf, Ben Liestman, Nafissatou Leye Diouf, Fatou Drame, Karleen Coly, Remy Serge Manzi Muhire, Safiatou Thiam, Papa Amadou Niang Diallo, Coumba Toure Kane, Cheikh Ndour, Erik Volz, Sharmistha Mishra, Stefan Baral, Peter Vickerman

**Affiliations:** ^1^ Population Health Sciences, Bristol Medical School University of Bristol Bristol UK; ^2^ Department of Infectious Disease Epidemiology Imperial College London UK; ^3^ Department of Epidemiology Johns Hopkins Bloomberg School of Public Health Baltimore MD USA; ^4^ Enda Sante Dakar Senegal; ^5^ Institut de Recherche en Santé de Surveillance Epidemiologique et de Formations Dakar Senegal; ^6^ St Michaels Hospital University of Toronto Toronto Canada; ^7^ Division de La Lutte Contre Le Sida et Les IST Ministry of Health Dakar Senegal; ^8^ Department of Health National AIDS Council of Senegal Dakar Senegal; ^9^ College of Engineering, Mathematics and Physical Sciences University of Exeter Exeter UK

**Keywords:** population attributable fraction, HIV, female sex workers, men who have sex with men, clients, condom use, key populations

## Abstract

**Introduction:**

Key populations including female sex workers (FSW) and men who have sex with men (MSM) bear a disproportionate burden of HIV. However, the role of focusing prevention efforts on these groups for reducing a country's HIV epidemic is debated. We estimate the extent to which HIV transmission among FSW and MSM contributes to overall HIV transmission in Dakar, Senegal, using a dynamic assessment of the population attributable fraction (PAF).

**Methods:**

A dynamic transmission model of HIV among FSW, their clients, MSM and the lower‐risk adult population was parameterized and calibrated within a Bayesian framework using setting‐specific demographic, behavioural, HIV epidemiological and antiretroviral treatment (ART) coverage data for 1985 to 2015. We used the model to estimate the 10‐year PAF of commercial sex between FSW and their clients, and sex between men, to overall HIV transmission (defined as the percentage of new infections prevented when these modes of transmission are removed). In addition, we estimated the prevention benefits associated with historical increases in condom use and ART uptake, and impact of further increases in prevention and treatment.

**Results:**

The model projections suggest that unprotected sex between men contributed to 42% (2.5 to 97.5th percentile range 24 to 59%) of transmissions between 1995 and 2005, increasing to 64% (37 to 79%) from 2015 to 2025. The 10‐year PAF of commercial sex is smaller, diminishing from 21% (7 to 39%) in 1995 to 14% (5 to 35%) in 2015. Without ART, 49% (32 to 71%) more HIV infections would have occurred since 2000, when ART was initiated, whereas without condom use since 1985, 67% (27 to 179%) more HIV infections would have occurred, and the overall HIV prevalence would have been 60% (29 to 211%) greater than what it is now. Further large decreases in HIV incidence (68%) can be achieved by scaling up ART in MSM to 74% coverage and reducing their susceptibility to HIV by two‐thirds through any prevention modality.

**Conclusions:**

Unprotected sex between men may be an important contributor to HIV transmission in Dakar, due to suboptimal coverage of evidence‐informed interventions. Although existing interventions have effectively reduced HIV transmission among adults, it is crucial that further strategies address the unmet need among MSM.

## 1 Introduction

Key populations (KP) such as female sex workers (FSW) and men who have sex with men (MSM) bear a disproportionate burden of HIV 1, 2, 3, 4, 5. The HIV‐associated vulnerabilities experienced by KP are thought to play an important role in the transmission of HIV in low HIV prevalence settings, but less so in settings with generalized HIV epidemics. Existing analyses suggest that FSW and their clients contribute less than 30% 1, 3 and 10% 6 of prevalent HIV infections among adults in sub‐Saharan Africa (SSA), respectively, although these analyses only considered the proportion of prevalent infections that were among clients or FSW. In addition, recent reviews of the UNAIDS Modes of Transmission (MOT) model 7, 8 suggest that the role of KP is small in SSA, with the cumulative percentage of new annual infections due to KP generally being less than 25%.

These estimations of the role of KP to HIV epidemics do not incorporate the full chain of transmission originating from KPs, such that a male who becomes infected by another male could then infect a female, resulting in the initial male not only contributing to the MSM HIV epidemic but also the heterosexual epidemic. Indeed, for commercial sex, these analyses conflict with emerging dynamic modelling analyses suggesting that >90% of all HIV transmissions to date are directly or indirectly due to sex work in the low‐to‐moderate prevalence HIV epidemics of Burkina Faso, Cote d'Ivoire and Benin, and over 65% in the higher‐prevalence HIV epidemic in Kenya 9, 10, 11, 12, 13, 14. These analyses challenge the conventional wisdom and existing epidemiological tools such as the MOT model. In contrast, two recent studies for Nigeria and Cote d'Ivoire suggest MSM contribute little (<10%) to these HIV epidemics 14, 15. This is despite MSM experiencing a high HIV burden 4, 16, 17 and engaging in sexual partnerships with women, so contributing bridging infections to the wider population 18. Limitations in existing evidence emphasizes the urgent need to improve our estimates of the contribution of KP to HIV epidemics in SSA; this basic epidemiological measure is crucial for prioritizing HIV programming.

Senegal has a low HIV prevalence among adults (0.4% in 2016) 19, thought to be due to a comprehensive response to the epidemic 20. Nevertheless, the HIV burden among KP is much higher (5.9% in FSW and 29.7% in MSM in 2016) 21, 22, 23, 24, 25, 26, 27. Despite the low prevalence of HIV in the adult population and high prevalence among KP, current estimates suggest that commercial sex and sex between men contribute little (<15%) to existing HIV transmission in Senegal 3, 8, although these estimations were limited as they did not incorporate the dynamic aspect of transmission.

To remedy these limitations, we undertook a dynamic model assessment of the contribution of commercial sex and sex between men to HIV transmission in Dakar, Senegal. We also estimated the impact of historical increases in the coverage of antiretroviral therapy (ART) and condom use among KP, and the potential impact of further uptake of prevention and treatment interventions.

## 2 Methods

### 2.1 Model description

**Figure 1:**
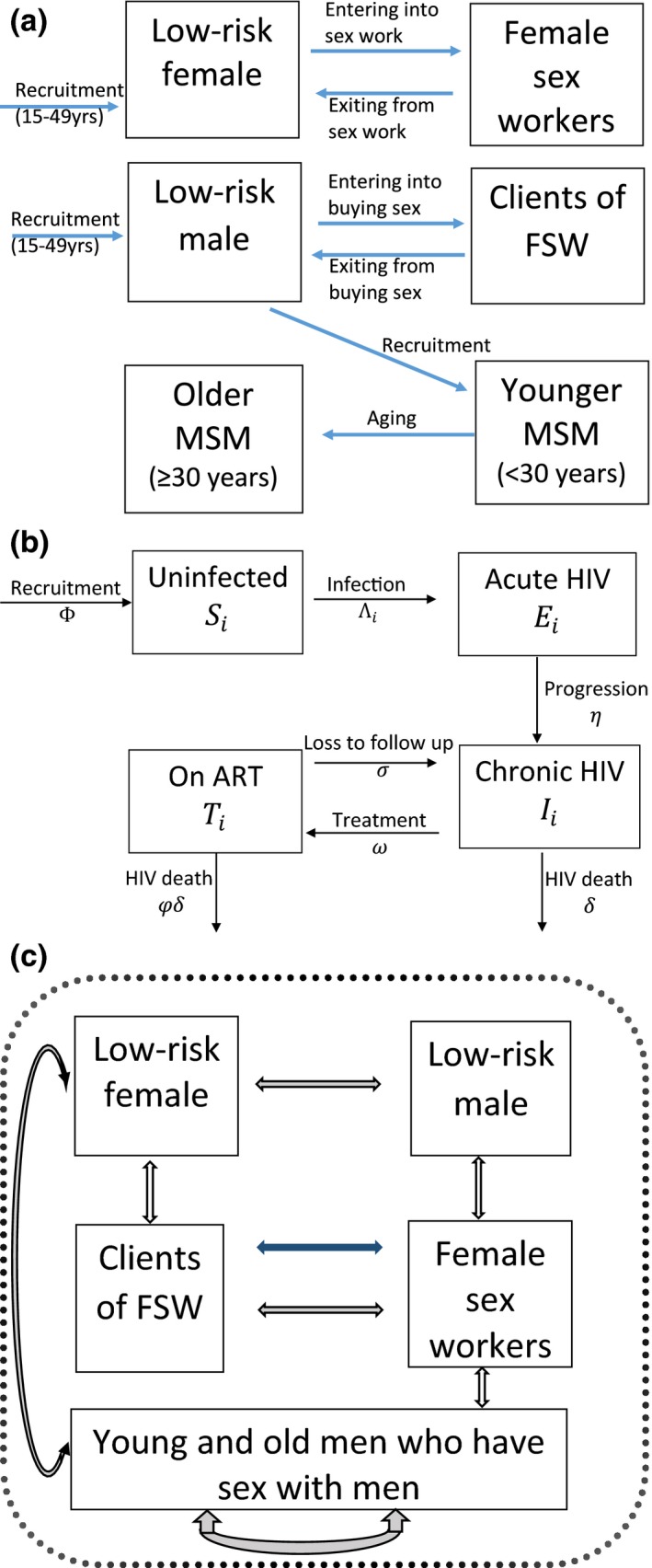
Model schematic illustrating the **(a)** movement of individuals in and out of different sub‐populations (male and female low‐risk, clients, female sex workers (FSW), younger and older men who have sex with men (MSM), **(b)** stratification of the population with respect to HIV infection and **(c)** sexual interactions which can result in HIV transmission among female, male, FSW, their clients and MSM. Blue arrow in Figure 1c shows commercial sex and all other arrows show sex with main and casual partners.

We developed a dynamic HIV transmission model to evaluate the extent to which FSW, clients of FSW (referred to as clients hereafter) and MSM contribute to the overall HIV epidemic in Dakar, Senegal. The model did not include people who inject drugs because of their low prevalence in Dakar (<0.07% of the adult population 28). The model considers adults (15 to 49 years), and divides the population into six sub‐populations: low‐risk females and males, clients, FSW, young MSM (<30 years) and old MSM (≥30 years) (Figure 1). Transgender women (TGW) were not explicitly included in the model because of insufficient data to parameterize this risk population. Low‐risk individuals are defined as people who are not MSM and do not report commercial sex.

Individuals enter the modelled population in the low‐risk groups when they become sexually active, at a rate that balances non‐HIV deaths and reflects population growth. Low‐risk males and females can become clients and FSW at specified rates, with both practising commercial sex for an average duration. Similarly, MSM transition from the low‐risk male population, and age from the young to the old MSM group, but remain as MSM until death.

The model captures HIV transmission among the sub‐populations through vaginal and anal sex (VI and AI, respectively) between all males and females, and AI within the MSM group (Figure 1). The model stratifies the population with respect to HIV infection and disease progression (Figure 1b). Upon infection, susceptible individuals acquire acute HIV infection before progressing to chronic infection. Chronically‐infected individuals experience HIV‐related mortality, but can also be recruited onto ART, which reduces HIV‐related mortality. Individuals on ART can be lost to follow up, whereupon they return to the chronic infection stage. All sub‐populations also experience non‐HIV‐related death.

The model incorporates HIV transmission due to main, casual and commercial sexual partnerships. Commercial partnerships occur between FSW and their clients, while main and casual partnerships between men only occur among MSM. All other heterosexual main and casual partnerships occur between all groups. The risk of HIV transmission for each individual is related to the HIV prevalence of their sexual partners, with the HIV transmission risk being elevated if they have acute infection, and reduced if they are on ART. Transmission risk is also related to the frequency of sex between different partnerships and is reduced through condom use. The consistency of condom use is time dependent and varies by type of partnership. The model assumes some males are circumcised, which reduces the risk of HIV acquisition. We assume heightened transmission risk in the initial stages of an HIV epidemic to capture the effects of risk heterogeneity. The model is described in Data S1.

### 2.2 Model parameterization and calibration

Recent model parameter and calibration data for FSW, clients and MSM were obtained from three integrated behavioural and biological assessment (IBBA) surveys undertaken in Dakar, Senegal, from 2015 to 2016 25 (client data are unpublished). Sexual behaviour data related to FSW, clients and MSM came from these surveys. The survey used to parameterize the MSM component of the model also included some TGW.

In addition, older IBBA surveys were used to determine whether risk behaviour has changed over time, and how the HIV epidemic in different risk groups has evolved. Importantly, this included trends in condom use for different risk groups. However, differences in the behavioural measures used made it difficult to evaluate how behaviours changed over time, and so uncertainty was incorporated into those trends. Adult population HIV‐related epidemiological and sexual behaviour data were obtained from the Demographic Health Surveys (DHS) for 2005 and 2010 29, 30. Table S1 gives a summary of IBBA surveys used in the modelling, while the model parameterization is summarized in Table 1 and included in full in Table S2.

**Table 1:** Parameters used for female sex workers (FSW), their clients and men who have sex with men (MSM) in 2016

Model parameter	FSW	Clients	Young MSM (<30 years)	Old MSM (≥30 years)
Size estimates (% of adult women or men)	0.5% (0.3 to 0.9%)	5.1% (1.8 to 12.1%)	1.2% (0.8 to 2.3%)	
HIV‐1 prevalence	5.9% (1.8 to 10.0%)	1.2% (0.5 to 2.4%)	28.6% (19.8 to 37.5%)^a^	37.0% (14.8 to 59.2%)
Level of viral suppression in individuals living with HIV	73.7% (60.9 to 84.2%)	NA	35.4% (23.9 to 48.2%)	
Frequency of partners per year				
Commercial (FSW/Clients)	516.4 (368.2 to 691.2)^b^	69.6 (62.4 to 78.0)	–	–
Main heterosexual	0.58 (0.47 to 0.73)	1.9 (1.3 to 2.6)	0.88 (0.39 to 1.5)	0.88 (0.39 to 1.5)
Casual heterosexual	0.96 (0.48 to 1.6)	0.6 (0.15 to 1.4)	2.3 (0.55 to 5.1)	2.3 (0.55 to 5.1)
Main (MSM sex)	–	–	0.71 (0.59 to 0.83)	0.71 (0.59 to 0.83)
Casual (MSM sex)	–	–	5.4 (2.5 to 9.2)	4.8 (1.3 to 9.2)
% of commercial sex acts that are anal^c^	4.1% (2.1 to 6.5%)	5.1% (3.8 to 6.5%)		
Frequency of vaginal sex acts per year				
Main heterosexual partners	98.3 (78.5 to 117.5)	97.3 (73.6 to 124.6)	65.3 (56.2 to 74.4)	65.3 (56.2 to 74.4)
Casual heterosexual partners	6.2 (4.2 to 8.2)	3.8 (2.1 to 5.9)	4.5 (2.6 to 6.4)	4.5 (2.6 to 6.4)
Frequency of anal sex acts per year				
Main heterosexual partners	9.9 (3.6 to 15.6)	6.6 (3.4 to 11.1)	5.2 (1.6 to 9.4)	5.2 (1.6 to 9.4)
Casual heterosexual partners	0.49 (0.12 to 1.2)	0.33 (0.097 to 0.75)	0.52 (0.04 to 1.0)	0.52 (0.04 to 1.0)
Frequency of anal sex for men with men per year (MSM sex)				
Main partnerships of MSM	–	–	105.6 (91.0 to 120.6)	105.6 (91.0 to 120.6)
Casual partnerships of MSM	–	–	7.6 (6.7 to 8.2)	15.2 (13.2 to 16.4)

NA denotes not available.

a Alternative IBBA survey from 2014 suggests lower HIV prevalence of 13.3% (9.7 to 17.4%).

b Uncertainty range widened based on different estimates from the 2016 survey and earlier surveys.

c The rest are vaginal.

**Figure 2:**
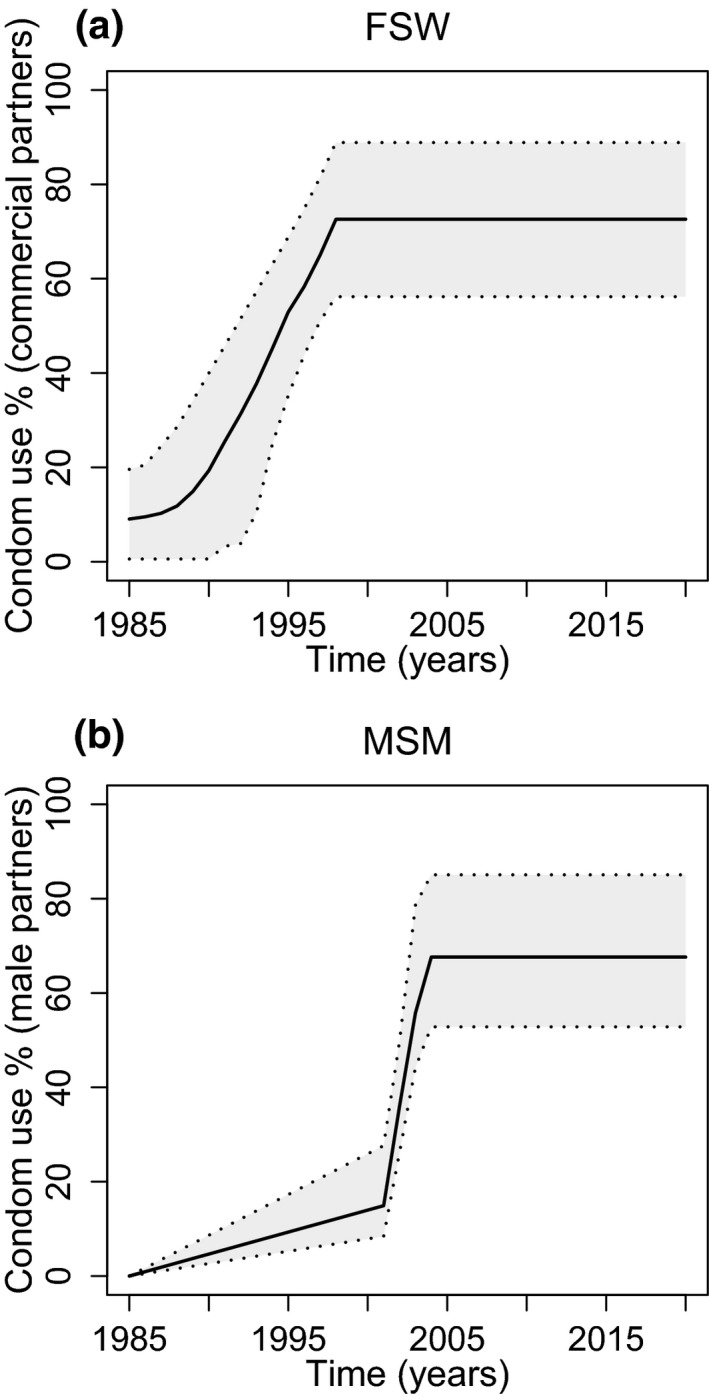
Modelled condom use trends for **(a)** female sex workers (FSW) and **(b)** men who have sex with men (MSM). **(a)** FSW condom use with commercial partners for vaginal intercourse. Assume condom use for anal intercourse is half that of vaginal intercourse for all years. **(b)** MSM condom use with regular and casual male partners. We assume some bias in reporting so all rates have been multiplied by a bias factor of 0.7 to 1.0 – lower bound of 0.7 chosen to give overall lower bound of 0.5, as seen in figure.

Based on data from numerous FSW and client IBBA surveys between 1985 and 2016 26, 31, 32, 33, 34, 35, 36, and national data on increases in condom distribution between 1988 and 1997 37, condom use during commercial VI sex between FSW and their clients (Figure 2a) was assumed to increase from negligible levels in 1985 33, up to a high stable level from 1998 onwards between 54 and 90% 24, 26, 34. This large range was based on the difference between client‐ and FSW‐reported condom use estimates in 2015 to 2016. Similarly, based on data from MSM IBBA surveys, condom use in last sex act for male partners of MSM was assumed to be negligible in 1985, low (10% to 30%) in 2001 38, up to 70 to 85% by 2003 to 2007 22, 39, 40 and constant thereafter (Figure 2b) (2016 survey). The Data S1 and Figure S1 include further details on the condom use assumptions.

Senegal population size estimates for 1980 to 2020 and gender‐specific death rates were obtained from UNDP 41, with population growth rates being fit to this data. Population size estimates for FSW and MSM were produced as part of the 2015 IBBA surveys using the service multiplier and unique object methods 42. The population size of clients was estimated through balancing the overall demand for commercial sex of FSW with that of clients (see Data S1).

ART data for the Senegal population came from the World Bank and UNAIDS 43, 44 (Figure S2), suggesting ART coverage increased from negligible levels in 2000 to 40% of individuals living with HIV in 2015. For FSW, these coverage trends were scaled up because the 2016 IBBA found 74% of FSW living with HIV were virally suppressed (unpublished data from 2016 FSW survey 25). ART recruitment rates were calibrated to give the trends in ART coverage. Other HIV biological parameters came from literature (Table S2).

**Figure 3:**
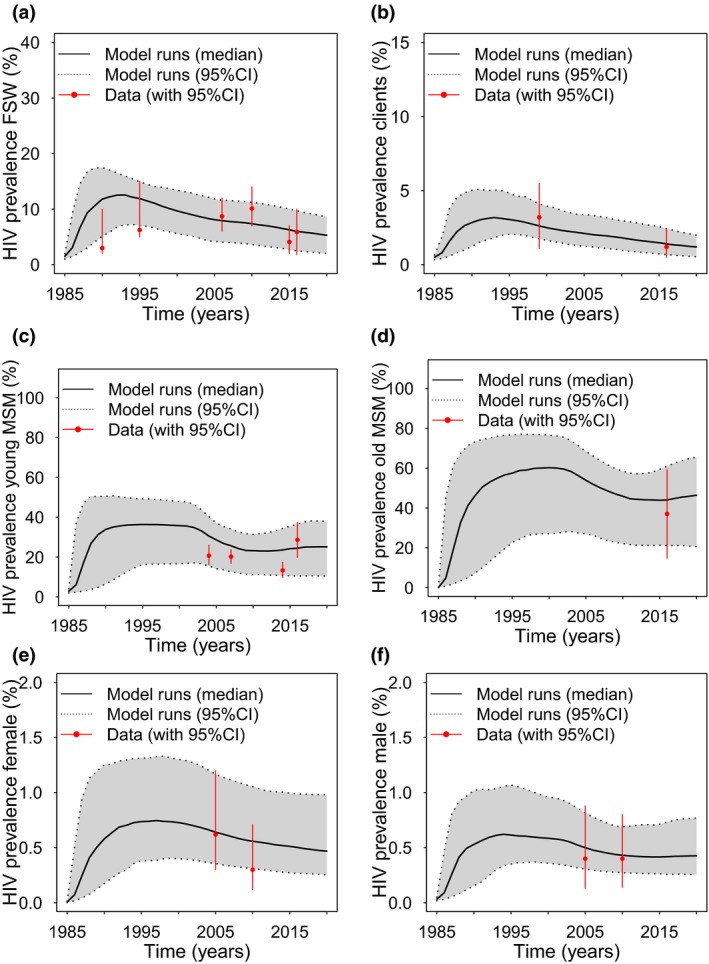
A comparison of model fits with HIV prevalence estimates from 1985 to 2020 for **(a)** female sex workers (FSW) **(b)** clients of FSW,** (c)** younger men who have sex with men (MSM) (<30 years old), **(d)** older MSM (≥30 years old) and **(e)** female and **(f)** male overall adult populations. Continuous black line shows median projections from all the model fits, with dashed lines and grey shaded areas showing 95% credibility intervals. Red points and lines show data with 95% confidence intervals.

Uncertainty ranges were assigned to most model parameters, with most parameters being fixed over time except for the rate of ART recruitment, levels of condom use and frequency of sex for MSM with their male partners, which data suggest increased from 2007 to 2016 21, 25. To incorporate uncertainty, 10,000 parameter sets were randomly sampled from their uncertainty ranges (Table S2 and Table 1). For each parameter set, the model was run while including temporal increases in condom use, ART coverage and the frequency of sex for male MSM partners. Any run producing HIV prevalence projections that agreed with early IBBA HIV prevalence data for FSW (1990 or 1995) and clients (1999) and recent HIV prevalence data for young MSM from 2014 to 2016 were selected as a model fit. These data suggested a HIV prevalence of 2.0 to 10.0% in 1990 and 5.0 to 15.0% in 1995 in FSW, 1.1 to 5.5% in clients (1999) and 9.7 to 37.7% in MSM, with these prevalence estimates shown in Figure 3 (data sources in Table S1). The wide range for MSM is due to contrasting estimates from two IBBA surveys in 2014 and 2016. Other HIV prevalence data for all sub‐groups are also shown in Figure 3. The model was not calibrated to these data, but instead the data were used to validate the accuracy of the model projections.

### 2.3 Analyses

#### 2.3.1 Contribution of commercial sex and sex between men to HIV transmission

To estimate the contribution of commercial sex between FSW and clients and sex between men to the overall HIV epidemic (referred to as population attributable fraction or PAF), model fits were used to estimate the proportion of new HIV infections that would be prevented by setting the transmission probability for commercial sex or sex between men to zero over a specific time period. This was estimated for 1 or 10 years from 1995, 2005 and 2015.

#### 2.3.2 Impact of existing interventions

Model fits were used to explore the likely impact of historical increases in ART coverage and condom use on the evolution of the HIV epidemic for different population sub‐groups. This was determined by re‐running the model fits, but with no ART and/or condom use.

#### 2.3.3 Impact of scaling up interventions

We then assessed the impact of increasing the coverage of ART among MSM from 2017 to 2030, such that the proportion of MSM living with HIV who are virally suppressed increases to the same coverage as FSW (74%) by 2020. This increase in ART coverage was also considered among low‐risk individuals. To capture the possible effect of introducing pre‐exposure prophylaxis for HIV (PrEP) and/or further increases in condom use, we also investigated the impact of an intervention that reduces an individual's average risk of becoming HIV positive. This was estimated by reducing by a third the susceptibility to HIV transmission among (i) MSM, (ii) FSW or (iii) all low‐risk individuals. This is roughly equivalent to putting 40% of MSM on PrEP (assuming 85% effectiveness 46, 47), 50% of FSW on PrEP (assuming 66% effectiveness 47, 48) or reducing the number of unprotected sex acts by 40% through increased condom use (assuming 82.5% effectiveness per sex act). We also assumed a scenario where both were achieved for MSM (PrEP and condom use), reducing susceptibility by two‐thirds.

#### 2.3.4 Uncertainty analysis

We performed linear regression analyses of covariance (ANCOVA) 49 to determine which parameters contribute most to the variability in the 10‐year PAF estimates for commercial sex and sex between men for 2015.

## 3 Results

### 3.1 Existing epidemiological insights

Two hundred model runs agreed with the HIV prevalence calibration data from FSW, clients and MSM. Despite only being calibrated to one‐third of the prevalence estimates (5/17 data points), these model fits agreed closely with observed HIV prevalence trends for all groups (Figure 3). These model fits suggest the HIV prevalence among FSW, clients and the adult population has been in steady decline since the mid‐90s, but may have increased recently among MSM.

### 3.2 Contribution of commercial sex and sex between men to HIV transmission

**Figure 4:**
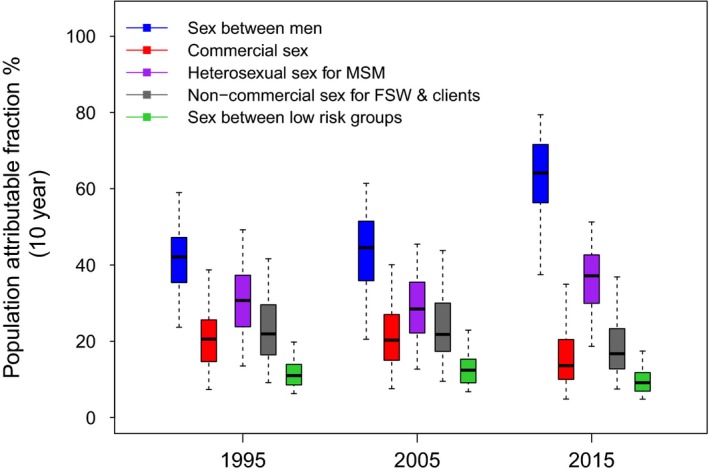
Ten‐year population attributable fraction (PAF) for sex between men (blue), commercial sex (red), heterosexual sex for men who have sex with men (MSM) (purple), non‐commercial sex for female sex workers (FSW) and their clients (grey), and sex between low‐risk groups (green). The PAF is estimated as the proportion of all HIV infections prevented over 10 years from 1995, 2005 or 2015 if the HIV transmission risk due to a specific type of sexual behaviour is removed. The box plots signify the uncertainty (middle line is median, limits of boxes are the 25% and 75% percentiles and whiskers are 2.5% and 97.5% percentile range) in the PAF estimates due to uncertainty in the model parameters.

For the 10 years from 1995, a median of 42.1% (2.5 to 97.5%, percentile range 23.7 to 59.0%) of new HIV infections could have been prevented (10‐year PAF) if the risk due to sex between men had been removed over this period, respectively, with this increasing to 64.1% (37.4 to 79.4%) by 2015 (Figure 4). The increase in the 10‐year PAF for sex between men in 2015 is due to the increase in the frequency of AI sex acts between MSM over this time period. Much of this effect of MSM is due to their heterosexual partnerships with females, such that removing this risk prevents 37.1% (18.7 to 51.3%) of HIV infections over 10 years from 2015 (Figure 4). In contrast, the 10‐year PAF for commercial sex was 20.6% (7.3 to 38.7%) and 13.6% (4.8 to 35.0%) for 1995 and 2015 respectively (Figure 4). For sex between men, one‐year PAFs were lower than 10‐year PAFs, but were similar for commercial sex (Table 2).

**Table 2:** Population attributable fraction for sex between men and commercial sex. The population attributable fraction is defined as the proportion of HIV infections prevented if the HIV transmission risk due to sex between men or commercial sex was removed from 1995, 2005 or 2015 for 1 or 10 years

	1995	2005	2015
PAF (1 year)			
Sex between men	36.2% (21.5% to 52.5%)	28.9% (13.3% to 42.8%)	51.4% (27.3% to 66.7%)
Commercial sex	19.8% (8.3% to 38.7%)	21.6% (8.8% to 36.9%)	13.8% (5.1% to 31.1%)
PAF (10 years)			
Sex between men	42.1% (23.7% to 59.0%)	44.6% (20.5% to 61.4%)	64.1% (37.4% to 79.4%)
Commercial sex	20.6% (7.3% to 38.7%)	20.3% (7.6% to 40.1%)	13.6% (4.8% to 35.0%)

The analysis of covariance showed that many parameters contributed to the uncertainty in the PAF estimates (Data S1).

### 3.3 Impact of existing interventions

**Figure 5:**
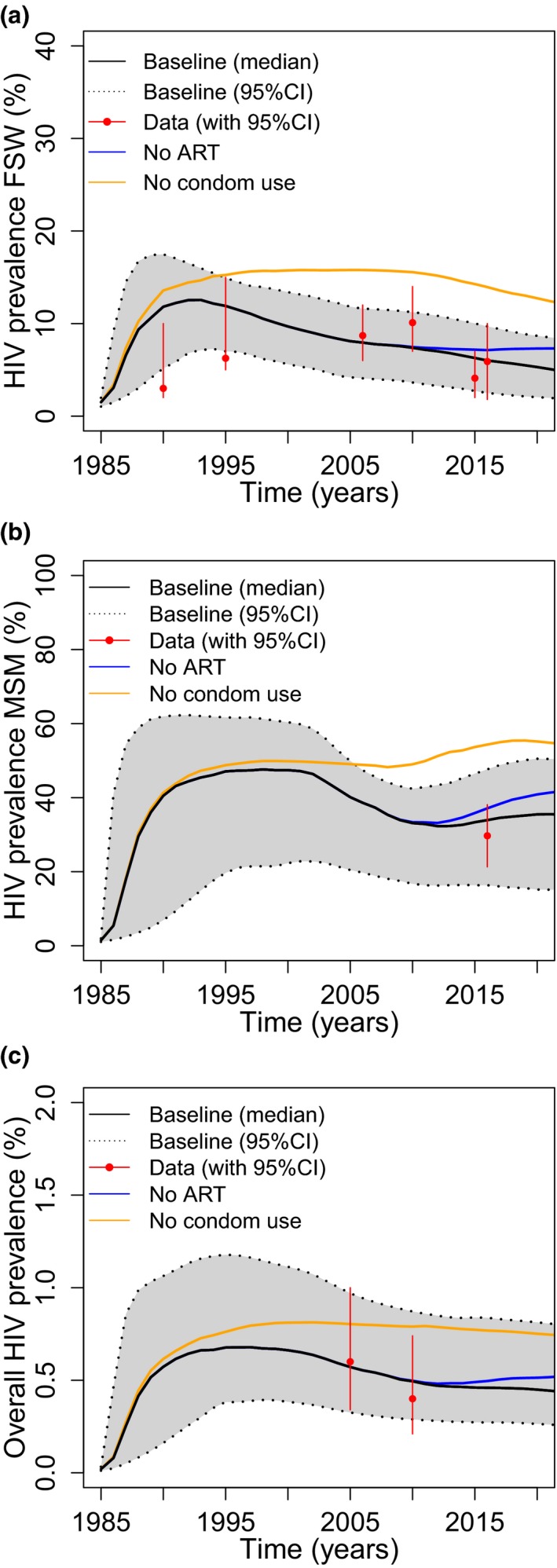
Projections of the impact of removing existing levels of antiretroviral therapy (ART) and condom use on HIV prevalence trends for **(a)** female sex workers (FSW), **(b)** all men who have sex with men (MSM) and **(c)** overall general population prevalence. Figures show baseline (median with 95% credibility intervals) trends, and median trends with no effect of ART (median‐blue) or no condom use (median‐orange).

Model projections (Figure 5) suggest that if there had been no ART scale‐up since 2000, in relative terms, the HIV prevalence in FSW in 2015 would have been 13.5% (6.1 to 23.8%) higher, 7.0% (4.0 to 11.0%) higher in MSM and 5.7% (2.2 to 10.2%) higher overall. In contrast, if condom use had not increased in the mid‐ to late‐1990s, the HIV prevalence in MSM would have been 56.6% (27.9 to 160.3%) higher in 2015, 2.1 times (1.5 to 6.8) higher in FSW and 60.2% (28.9 to 210.9%) higher in the general population (Figure 5). In terms of new HIV infections, without ART scale‐up, 49.0% (32.3 to 71.3%) more HIV infections would have occurred in Dakar since 2000 when ART was introduced, whereas without any condom use since the mid‐1980s, 66.5% (27.2 to 178.5%) more HIV infections would have occurred.

### 3.4 Impact of scaling up interventions

**Figure 6:**
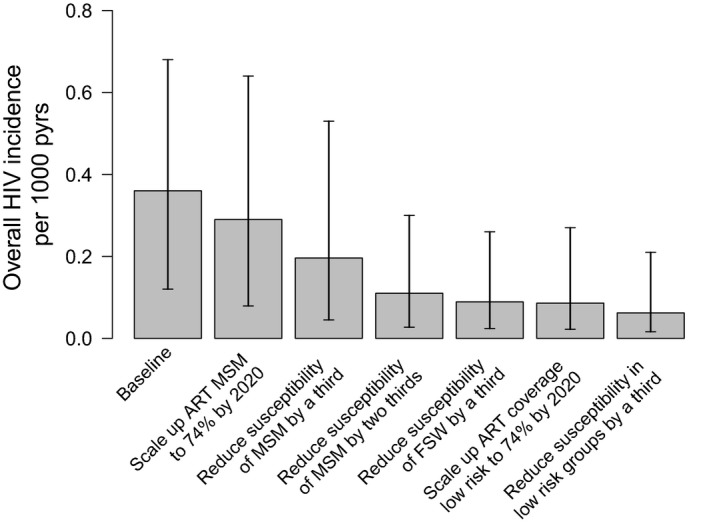
Effect of changes in antiretroviral therapy (ART) coverage or susceptibility to infection (due to pre‐exposure prophylaxis (PrEP) or increases in condom use) from 2017 to 2030 on overall projected HIV incidence per 1000‐person years in 2030. The plots show median and 95% credibility intervals of the projections. Scenarios included are additive on top of the previous scenarios from left to right, firstly including baseline projections, then projections with scale‐up of ART coverage in men who have sex with men (MSM) from 2017 such that 74% of MSM living with HIV are virally suppressed by 2020 (same as female sex workers (FSW)), reduce susceptibility of MSM by 33% and then 66% (similar to increase condom use or PrEP coverage in MSM), reducing susceptibility of FSW by 33%, scale‐up ART coverage in low risk such that 74% are virally suppressed by 2020 and reduce susceptibility of low‐risk groups by 33%.

Scaling up ART among MSM such that 74% are virally suppressed by 2020 (35.4% of MSM living with HIV are currently virally suppressed) would decrease the overall HIV incidence in Dakar by 14.7% (4.9 to 47.1%) by 2030 (Figure 6). If the HIV susceptibility in MSM is also reduced by a third then incidence would decrease by 43.9% (14.9 to 76.0%), while it decreases by 68.3% (42.0 to 88.6%) if susceptibility reduces by 66%. This captures what could be achieved from putting 40% of HIV‐negative MSM on PrEP and reducing the number of non‐condom protected sex acts by 40%. However, further decreases in FSW or general population susceptibility (by a third) or increases in ART coverage in the general population (so 74% are virally suppressed) will not add much more impact, with these interventions in combination decreasing the overall incidence by 82.4% (62.4 to 93.3%). This is due to the small contribution of these groups to HIV transmission in Dakar.

## 4 Discussion

Our findings suggest that unprotected anal sex between men, both through individuals who identify as cis‐male and TGW, may be the main contributor to the HIV epidemic in Dakar, Senegal, with up to 64% of new HIV infections being preventable if the HIV prevention and treatment needs of these individuals could be met. Conversely, unlike other low HIV prevalence settings in SSA, much fewer (<15%) HIV infections are attributable to commercial sex between FSW and their clients 10, 11, 14. The greater role of MSM in Dakar is partly due to their greater number compared to FSW (1.2% of adult males compared to 0.5% of adult females in 2015) and much higher HIV prevalence (29.7% for MSM compared to 6.6% for FSW in 2016), with this disparity being due to long‐term prevention activities among FSWs in Dakar, but not MSM. In addition, the heterosexual activity of MSM is also important; stopping this mode of transmission could prevent 37% of new HIV infections from 2015 to 2025.

These results are useful for planning intervention strategies going forward, with most impact being achieved from expanding interventions among MSM, including cis‐male and TGW. These data suggest that increasing ART provision to MSM could reduce HIV incidence by 15% if the proportion of MSM living with HIV who are virally suppressed doubled from 35% to 74%. In addition, reducing their susceptibility to HIV infection could also have important benefits, with the successful introduction of PrEP among MSM possibly reducing HIV incidence by a further 30 percentage points, and an expansion of condom use having similar benefits. Such interventions, on their own and in combination with ART scale‐up have been shown to be effective 46, 50, 51, 52, 53, cost‐effective^54^, ^55^ and feasible among MSM, and so their expansion should not be delayed. As same‐sex practices remain criminalized in Senegal, and MSM face discrimination 56, it is also important that effective stigma mitigation interventions are combined with these interventions to ensure their effectiveness 25.

Our findings suggest little impact is achieved from reducing risk or increasing ART coverage among other population sub‐groups, including FSW, further emphasizing the need to focus new interventions on MSM. However, this is contingent on sustained high levels of condom use and ART uptake among FSW. That is, our results do not suggest that efforts should be transferred from existing interventions to refocus on MSM interventions, but rather that additional efforts are focused on MSM. Indeed, our projections emphasize existing interventions have had considerable impact, halving HIV prevalence compared to what it could have been in 2017.

## 5 Strengths and limitations

Strengths of our analysis include the use of detailed setting‐specific data from numerous bio‐behavioural surveys among FSW, their clients and MSM from 1985 to 2015, as well as two general population surveys from 2005 and 2010. In addition, the inclusion of MSM in our modelling improves on previous models that have frequently ignored MSM based on assumptions of decreased relevance across Sub‐Saharan Africa 57, and rarely included them in dynamic PAF estimates 9, 58. Lastly, another key strength in our analyses is the accuracy of our model projections compared to data that were not fitted, which included two‐thirds of available HIV prevalence estimates.

Despite using best available data, this model has several limitations. It did not incorporate any commercial sex among MSM due to limited data. Fortunately, including commercial sex among MSM would not have changed our general results since the HIV infections would still be attributed to same‐sex practices. The model also did not explicitly include TGW because of insufficient data to do so. However, the 2016 MSM survey used to parameterize and calibrate the model included some TGW, as did the MSM size estimation data used by the model, and so their contribution was captured to an extent by the model. Moving forward, these analyses and associated bio‐behavioural surveys need to better assess gender identity to enable a better assessment of their role in HIV transmission. The modelling was also limited by uncertainty in many model parameters. In addition, differences in the condom use and behavioural measures used by studies made it hard to evaluate temporal changes in risk. To account for these uncertainties, we associated wide ranges to all uncertain parameters and trends in condom use and risk behaviours, and used Bayesian fitting methods to account for and constrain this uncertainty through calibrating to HIV prevalence data. Importantly, our findings were robust to this uncertainty.

Another limitation of our analysis was the relative simplicity of the model with respect to HIV natural history, the portrayal of ART and heterogeneity in sexual behaviours among each sub‐population. Although greater detail has been included in other models, there is no consensus on the appropriate level of complexity for specific models. One specific simplification is not stratifying MSM by whether they normally have insertive or receptive anal sex. This was done to avoid overly complicating the model, which should not have affected our results because they have the same sexual behaviour with females; the main indirect mechanism for MSM contributing to overall HIV transmission.

Lastly, the model only considered Dakar. Although this limits the generalizability of the findings, it does increase the likely precision of the modelling because we did not make generalizing assumptions to produce an average portrayal of the Senegal epidemic. Despite the limited scope of the analysis, it is still likely that the results are relevant to the whole of Senegal because the HIV prevalence in MSM is generally high and increasing, while it is lower and decreasing among FSW. In addition, our analyses are also likely relevant to other West African settings with growing epidemics in MSM, similar or lower HIV prevalence in FSW 3, 4, and similar reporting of heterosexual sex among MSM 59, 60, 61.

## 6 Conclusion and implications

Previous analyses have shown a large role for commercial sex in some sub‐Saharan HIV epidemics 10, 11, 12, 13, 14, but this is the first to suggest that MSM, including individuals who identify as cis‐male and TGW, could also be having an important role in some lower‐ and middle‐income country settings, where other analyses have suggested a small role 14, 15, 62. This is likely due to the different behavioural and epidemiological situation among MSM in Dakar compared to these other settings. Although existing interventions have been successful in reducing HIV transmission, including early increases in condom use and recent scale‐up of ART among FSW, public health efforts are now needed to address the ongoing unmet need among MSM. This unmet need is resulting in MSM in Senegal and other settings in SSA experiencing uncontrolled HIV epidemics with high prevalence 57, which is driving HIV transmission in this setting and could be elsewhere. Scaling up prevention and treatment interventions for MSM should now be a high priority, with these initiatives needing to be sensitive to the legal context, and associated stigmas or discrimination that MSM experience. Without this policy shift, the HIV epidemics among MSM in Senegal and elsewhere in SSA are unlikely to decrease.

## Competing interests

The authors have no competing interests.

## Authors’ contributions

PV, MCB, SB, SM and SS conceptualized the study. CM performed the model analyses and wrote the first draft of the manuscript with PV. CM and LD developed the model and reviewed the literature for data sources for the model, helped by the Senegal and JHU team. PV supervised the model analyses. SB, SS, DD, CL, BL, NLD, FD, KC, RSMM, ST, PAND, CT and CN collected or contributed data for the modelling. JW and LD undertook data analyses for parameterizing and calibrating the model. All authors contributed to data interpretation, writing the manuscript and approved the final version.

## Supporting information


**Data S1.** Supplementary materials.
**Table S1**. Details of surveys used to parameterise and calibrate the model for different risk groups in Dakar
**Table S2. (a)** Demographic, sexual and behavioural parameters of female sex workers, clients, MSM and low risk populations. **(b)** HIV epidemiological parameters
**Figure S1**. Modelled condom use trends for female sex workers (FSW) and men who have sex with men (MSM). **(a)** FSW condom use with commercial partners for vaginal intercourse. Assume condom use for anal intercourse (AI) is half that of vaginal intercourse (VI) for all the years. **(b)** FSW condom use with main partners for VI. Condom use for anal intercourse (AI) with main partners is assumed to be 0.6 to 1.0 times that for VI. FSW condom use with casual partners for VI is 1 to 1.5 times that for main partners VI; AI with casual partners is 0.6 to 1.0 times that for VI with casual partners. **(c)** MSM condom use with male regular and casual partners. Assume some bias in reporting so all rates are multiplied by a bias factor of 0.7 to 1.0 – lower bound of 0.7 chosen to give overall lower bound of 0.5. **(d)** MSM condom use with female main and casual partners. Condom use is assumed to be the same for VI and AI.
**Figure S2.** ART coverage in Senegal from 2000 from UNAIDS AIDS info^[44]^ and World Bank^[43]^
**Figure S3**. Analysis of covariance results of parameters that contribute more than 4% variability to the 2015 **(a)** 10‐year commercial sex PAF and **(b)** 10‐year MSM PAF.
