# Supplementary material for: Estimating the contribution of key populations towards the spread of HIV in Dakar, Senegal
Source: J Int AIDS Soc. 2018 Jul 22;21(Suppl Suppl 5):e25126. doi: 10.1002/jia2.25126 (PMC6055131; doi:10.1002/jia2.25126)
Supplement: Supplementary file 1 — Data S1. Supplementary materials. Table S1. Details of surveys used to parameterise and calibrate the model for different risk groups in Dakar Table S2. (a) Demographic, sexual and behavioural parameters of female sex workers, clients, MSM and low risk populations. (b) HIV epidemiological parameters Figure S1. Modelled condom use trends for female sex workers (FSW) and men who have sex with men (MSM). (a) FSW condom use with commercial partners for vaginal intercourse. Assume condom use for anal intercourse (AI) is half that of vaginal intercourse (VI) for all the years. (b) FSW condom use with main partners for VI. Condom use for anal intercourse (AI) with main partners is assumed to be 0.6 to 1.0 times that for VI. FSW condom use with casual partners for VI is 1 to 1.5 times that for main partners VI; AI with casual partners is 0.6 to 1.0 times that for VI with casual partners. (c) MSM condom use with male regular and casual partners. Assume some bias in reporting so all rates are multiplied by a bias factor of 0.7 to 1.0 – lower bound of 0.7 chosen to give overall lower bound of 0.5. (d) MSM condom use with female main and casual partners. Condom use is assumed to be the same for VI and AI. Figure S2. ART coverage in Senegal from 2000 from UNAIDS AIDS info[44] and World Bank[43] Figure S3. Analysis of covariance results of parameters that contribute more than 4% variability to the 2015 (a) 10‐year commercial sex PAF and (b) 10‐year MSM PAF. [file JIA2-21-e25126-s001.docx]

**Supplementary materials**

**Overview model description**

We developed a dynamic HIV transmission model to evaluate the extent to which FSW, clients of FSW (referred to as clients hereafter) and MSM contribute to the overall HIV epidemic in Dakar, Senegal. The model considers adults (15-49 years), and divides the population into six sub-populations: low-risk females ($i=1$) and males $(i=2$), clients ($i=3$), FSW ($i=4$), young MSM ($<30 years, i=5$) and old MSM ($\geq30 years, i=6$) (Figure 1 in main text shows the model schematic). Low-risk individuals are defined as people that are not MSM and do not report commercial sex.

Individuals enter the modelled population when they become sexually active, at a rate $\Phi$ that balances non-HIV deaths and reflects population growth, with a proportion $p$ entering into the low-risk female group and the remainder $(1-p)$ entering the low-risk male group. Low-risk females become FSW at a rate $\kappa$ and stay as FSW for duration $1/\gamma$ years. Similarly, clients and MSM transition from the low-risk male population at rates $k$ and $\chi$, respectively. Clients remain as clients for a period of $1/g$ years before returning to the low-risk male group. Conversely, MSM move from the young to the old MSM group at a rate $\zeta,$ where they remain until death.

The model captures HIV transmission among the sub-populations through vaginal and anal sexual intercourse (VI and AI, respectively) between all males and females, and AI within the MSM group (Figure 1 in main text). The model stratifies the population with respect to HIV infection and disease progression such that for each sub-population$i$, there is uninfected ($S_{i}$), acute infection ($E_{i}$), chronic infection ($I_{i}$) and on ART ($T_{i}$), with $i=1,2...6$. Upon infection, susceptible individuals move to the acute stage of HIV infection, and stay there for a duration of $1/\eta$ before progressing to the chronic stage. In the chronic stage, individuals can experience HIV-related mortality at a rate $\delta$, or are recruited onto ART at a rate $\omega$ (time dependent). While on ART, HIV-related mortality is reduced by a factor $\varphi$ compared to what it was in the chronic stage. Individuals on ART can be lost to follow-up at a rate $\sigma$, whereupon they return to the chronic infection stage. All sub-populations also experience non-HIV related death at rates $\mu_{a}$ and $\mu_{b}$ for females and males, respectively.

The model incorporates HIV transmission due to main, casual and commercial sexual partnerships. Commercial partnerships can only occur between FSW and their clients, while main and casual partnerships between men only occur amongst the two MSM groups. All other main and casual partnerships between male and females can occur between individuals from all groups, including MSM. The risk of HIV transmission for a particular individual is related to the HIV prevalence of their sexual partners, with the HIV transmission risk being elevated by a factor $\nu$ if they are in the acute stage of infection, and reduced by a factor $\alpha$ if they are on ART, all compared to the transmission risk for the chronic stage. Transmission risk is also related to the average frequency of sex acts (denoted by ${\Psi_{ijh}}^{k})$ for different types of partnerships and between risk groups $i$ and $j$ (with ${\Psi_{ijh}}^{k}={\Psi_{jih}}^{k}$ to ensure they balance) , where $k$ denotes the type of sexual partner (main, casual or commercial) and $h$ denotes the type of sexual act (VI or AI). HIV transmission is reduced through condom use by a factor $\left( 1-\varepsilon\pi_{ijh}^{k} \right),$ where $\varepsilon$is the efficacy of condom use and $\pi_{ijh}^{k}$ is the average consistency of condom use reported by those in risk groups $i$ and $j$ (with $\pi_{ijh}^{k}=\pi_{jih}^{k}$ to ensure they balance). The consistency of condom use is assumed to be time dependent and varies depending on the type of partnership. The model assumes a proportion $\xi$ of males are circumcised, with the model assuming these males have a reduced risk of HIV acquisition, modelled by a factor (1-$\xi\vartheta$), where $\vartheta$ is the efficacy of circumcision. As described below, we assume heightened transmission risk in the initial stages of an HIV epidemic to capture the effects of risk heterogeneity. More details on the model and its equations are below.

**Model Equations**

**Force of infection**

Let $\rho_{j}^{k}$ be the probability of heterosexual mixing of a population group with group $j$ for each type of sexual partnership $k$ (main or casual partnerships), and $j$=1, 2, 3, 4, 5, and 6 denotes the sub-groups low-risk female, low-risk male, clients, FSW, young and old MSM respectively. If the subscripts $v$ and $a$ denote VI and AI, respectively, then in general the force of infection (FOI) $\Lambda_{i}^{k}$for risk group $i$ due to their $k$ partnership with groups j is:

$$\Lambda_{i}^{k}=n_{i}^{k}\sum_{relevant j} {(\beta}_{rv}\left( 1-\varepsilon\pi_{ijv}^{k} \right)\Psi_{ijv}^{k}+\beta_{ra}\left( 1-\varepsilon\pi_{ija}^{k} \right)\Psi_{ija}^{k})\rho_{j}^{k}B_{j}$$

Where $\beta_{rv}$ and $\beta_{ra}$ are the HIV transmission probability per sex act through VI and AI respectively which is gender specific ($r$=$x$for males to females or $y$for females to males), and the frequency of sex partners of type $k$ for risk group $i$is given by $n_{i}^{k}$. $B_{j}$is the HIV prevalence of the population they are having sex with, which also accounts for the cofactors that increase or decrease HIV transmission risk due to the HIV acute phase $E_{j}$ ($\nu)$, or if on HIV treatment $T_{j}$ ($\alpha_{j})$, such that $B_{j}=(\nu E_{j}+I_{j}+(1-\alpha_{j})T_{j})/N_{j}$, where $N_{j}$ is the total population for each sub-group $j$.

The probability of mixing with different population groups j for partnership type k, where j=1..6 , is $\rho_{j}^{k}$ where:

| Probability of mixing with **low risk females** for heterosexual main and casual partnerships | $\rho_{1}^{k}=\frac{n_{1}^{k}N_{1}}{n_{1}^{k}N_{1}+n_{4}^{k}N_{4}}$ |
| --- | --- |
| Probability of mixing with **low risk males** for heterosexual main and casual partnerships | $\rho_{2}^{k}=\frac{n_{2}^{k}N_{2}}{n_{2}^{k}N_{2}+n_{3}^{k}N_{3}+n_{5}^{k}N_{5}+n_{6}^{k}N_{6}}$ |
| Probability of mixing with **clients of FSW** for heterosexual main and casual partnerships | $\rho_{3}^{k}=\frac{n_{3}^{k}N_{3}}{n_{2}^{k}N_{2}+n_{3}^{k}N_{3}+n_{5}^{k}N_{5}+n_{6}^{k}N_{6}}$ |
| Probability of mixing with **FSW** for heterosexual main and casual partnerships | $\rho_{4}^{k}=\frac{n_{4}^{k}N_{4}}{n_{1}^{k}N_{1}+n_{4}^{k}N_{4}}$ |
| Probability of mixing with **young** **MSM** for heterosexual main and casual partnerships | $\rho_{5}^{k}=\frac{n_{5}^{k}N_{5}}{n_{2}^{k}N_{2}+n_{3}^{k}N_{3}+n_{5}^{k}N_{5}+n_{6}^{k}N_{6}}$ |
| Probability of mixing with **old** **MSM** for heterosexual main and casual partnerships | $\rho_{6}^{k}=\frac{n_{6}^{k}N_{6}}{n_{2}^{k}N_{2}+n_{3}^{k}N_{3}+n_{5}^{k}N_{5}+n_{6}^{k}N_{6}}$ |

These mixing equations assume that all sexual mixing for heterosexual casual and main partnerships is random based on the total number of sexual partnerships that each group provides.

For females, we allow the sexual behaviour of males with females to determine who the females have sex with and how many partners they have. Therefore, the total number of sexual partners of type k that males have with females in group ($i$) is:

$$\rho_{i}^{k}\sum_{l=2,3,5,6} n_{l}^{k}N_{l}$$

Where we have summed up all male partnerships of type k and seen how many of these will be with women of type $i$ ($i$=1 or 4). Note, here it is important to emphasise that $\rho_{i}^{k}$ is defined in terms of the self-reported frequency of partnerships by women, but this is then used to define an adjusted frequency of sexual partnerships that will balance the number reported by males. Therefore, if there are $N_{i}$ women in that group then each woman in the group has the following adjusted frequency of male sexual partners of type k:

$$\frac{\rho_{i}^{k}}{N_{i}}\sum_{l=2,3,5,6} n_{l}^{k}N_{l}$$

And so, the adjusted number of partners each woman in group $i$ has with men in each group ($j$) is

$$\frac{\rho_{i}^{k}}{N_{i}}\rho_{j}^{k}\sum_{l=2,3,5,6} n_{l}^{k}N_{l}.$$

We find this simplifies to the following when we substitute the formulation for $\rho_{j}^{k}$:

$$\frac{\rho_{i}^{k}}{N_{i}}n_{j}^{k}N_{j}$$

We also incorporate an additional term $\lambda_{i}$ that allows the FOI to be elevated by factor $w$ (> 1) at low prevalence, but tend to have the same endemic HIV prevalence, to incorporate the effect of heterogeneity increasing transmission in the initial stages of an HIV epidemic or at lower HIV prevalences. To do this we define $\lambda_{i}$as follows

$$\lambda_{i}=we^{\frac{P_{i}(t)}{P_{i}^{\infty}}ln(\frac{1}{w})}$$

Where $P_{i}(t)$ is prevalence of HIV in population group $i$at time $t$ and $P_{i}^{\infty}$ is the prevalence at endemic level in that population group.

Therefore, the FOI $\Lambda_{i}^{k}$ for the low-risk female population due to their main ($k=m$) and casual ($k=c$) partners is adapted as follows:

$$\Lambda_{1}^{k}=\lambda_{1}\frac{\rho_{1}^{k}}{N_{1}}\sum_{j=2,3,5,6} {[\beta}_{xv}\left( 1-\varepsilon\pi_{1jv}^{k} \right)\Psi_{1jv}^{k}+\beta_{xa}\left( 1-\varepsilon\pi_{1ja}^{k} \right)\Psi_{1ja}^{k}]n_{j}^{k}N_{j}B_{j}$$

FOI for the low-risk male population due to their main $(k=m$) and casual ($k=c$) partners is

$$\Lambda_{2}^{k}=\lambda_{2}{(1-\vartheta\xi)n}_{2}^{k}\sum_{j=1 or 4} {[\beta}_{yv}\left( 1-\varepsilon\pi_{2jv}^{k} \right)\Psi_{2jv}^{k}+\beta_{ya}\left( 1-\varepsilon\pi_{2ja}^{k} \right)\Psi_{2ja}^{k}]\rho_{j}^{k}B_{j}$$

Data from the recent client IBBA survey in 2016 found that clients report 45.4% (41.3-49.5%) of their main partners and 87.8% (83.1-93.4%) of their casual partners are FSW. We assume these partnerships with FSW are accounted for in their commercial partnerships because the client survey asked how many sex workers they had sex with in the last time period, irrespective of whether they paid for them. We allot the remaining partnerships that are not knowingly with FSW to be with either FSW or low risk female randomly – these are incorporated in to the clients adjusted frequency of main and casual partners $n_{3}^{m}$and $n_{3}^{c}$, respectively. The force of infection for clients due to their main, casual ($k=m,c$) and commercial partners are given as:

$$\Lambda_{3}^{k}=\lambda_{3}(1-\vartheta\xi)n_{3}^{k}\sum_{j=1 or 4} {[\beta}_{yv}\left( 1-\varepsilon\pi_{3jv}^{k} \right)\Psi_{3jv}^{k}+\beta_{ya}\left( 1-\varepsilon\pi_{3ja}^{k} \right)\Psi_{3ja}^{k}]\rho_{j}^{k}B_{j}$$

$$\Lambda_{3}^{co}=\lambda_{3}n_{3}^{co}{[\beta}_{yv}\left( 1-\varepsilon\pi_{34v}^{co} \right)(1-p_{a}^{co})+\beta_{ya}\left( 1-\varepsilon\pi_{34a}^{c} \right)p_{a}^{co}](1-\vartheta\xi)B_{4},$$

Where $p_{a}^{co}$ is the proportion of commercial sex acts that are anal.

The FOI for female sex workers is similarly

$$\Lambda_{4}^{k}=\lambda_{4}\frac{\rho_{4}^{k}}{N_{4}}\sum_{j=2,3,5,6} {{[\beta}_{xv}\left( 1-\varepsilon\pi_{4jv}^{k} \right)\Psi_{4jv}^{k}+\beta_{xa}\left( 1-\varepsilon\pi_{4ja}^{k} \right)\Psi_{4ja}^{k}]n}_{j}^{k}N_{j}B_{j}$$

$$\Lambda_{4}^{co}=\lambda_{4}{n_{4}^{co}[\beta}_{xv}\left( 1-\varepsilon\pi_{43v}^{co} \right)(1-p_{a}^{co})+\beta_{xa}\left( 1-\varepsilon\pi_{43a}^{c} \right)p_{a}^{co}]B_{3}$$

Men who have sex with men are assumed to have main and casual partnerships with women from the low risk female population and FSWs as well as other MSM. The FOI for young MSM due to their main and casual ($k=m,c$) partnerships with females is:

$$\Lambda_{5}^{k}=\lambda_{5}(1-\vartheta\xi)n_{5}^{k}\sum_{j=1 or 4} {[\beta}_{yv}\left( 1-\varepsilon\pi_{5jv}^{k} \right)\Psi_{5jv}^{k}+\beta_{ya}\left( 1-\varepsilon\pi_{5ja}^{k} \right)\Psi_{5ja}^{k}]\rho_{j}^{k}B_{j}$$

For MSM sexual intercourse with their male sexual partners, we denote the receptive AI and insertive AI HIV transmission probability as $\beta_{jrec}$ and $\beta_{jins}$ respectively where $j=5,6$ and subscript *rec* and *ins* are for receptive and insertive anal intercourse between MSM. The asterisk (*) show parameters related to MSM with their male partners. Then, the FOI for young MSM due to their main and casual ($k=m,c$) partnerships with other MSM is:

$$\Lambda_{5*}^{k}={\lambda_{5}(\beta}_{5rec}+\beta_{5ins})(1-\vartheta\xi)n_{5*}^{k}\sum_{j=5 or 6} \left( 1-\varepsilon\pi_{5j*}^{k} \right){(\Psi}_{5j*}^{k}/2)\rho_{j*}^{k}B_{j}$$

$$.$$

Where $\rho_{5*}^{k}$is the probability of mixing to form MSM male sexual partnerships with young or old MSM, and is given by $\rho_{5*}^{k}=n_{5*}^{k}N_{5}/(n_{5*}^{k}N_{5}+n_{6*}^{k}N_{6})$ and $\rho_{6*}^{k}=n_{6*}^{k}N_{6}/(n_{5*}^{k}N_{5}+n_{6*}^{k}N_{6})$

Similarly, the FOI for old MSM due to their female main and casual ($k=m,c$) partners

$$\Lambda_{6}^{k}=\lambda_{6}(1-\vartheta\xi)n_{6}^{k}\sum_{j=1 or 4} {[\beta}_{yv}\left( 1-\varepsilon\pi_{6jv}^{k} \right)\Psi_{6jv}^{k}+\beta_{ya}\left( 1-\varepsilon\pi_{6ja}^{k} \right)\Psi_{6ja}^{k}]\rho_{j}^{k}B_{j}$$

And due to their main and casual ($k=m,c$) partnerships with other MSM

$$\Lambda_{6*}^{k}={\lambda_{6}(\beta}_{6rec}+\beta_{6ins})(1-\vartheta\xi)n_{6*}^{k}\sum_{j=5 or 6} {\left( 1-\varepsilon\pi_{6j*}^{k} \right){(\Psi}_{6j*}^{k}/2)\rho}_{j*}^{k}B_{j}$$

The model equations are as follows.

**Model equations**

Low-risk female population

$$\frac{dS_{1}}{dt}=\Phi p+\gamma S_{4}-{(\Lambda}_{1}^{m}+\Lambda_{1}^{c})S_{1}-(\kappa+\mu_{1})S_{1}$$

$$\frac{dE_{1}}{dt}={(\Lambda}_{1}^{m}+\Lambda_{1}^{c})S_{1}+\gamma E_{4}-(\kappa+\eta+\mu_{1})E_{1}$$

$$\frac{dI_{1}}{dt}=\eta E_{1}+\sigma T_{1}+\gamma I_{4}-(\kappa+\omega+\delta+\mu_{1})I_{1}$$

$$\frac{dT_{1}}{dt}=\omega I_{1}+\gamma T_{4}-(\kappa+\sigma+\varphi\delta+\mu_{1})T_{1}$$

Low-risk male population

$$\frac{dS_{2}}{dt}=\Phi(1-p)+gS_{3}-{(\Lambda}_{2}^{m}+\Lambda_{2}^{c})S_{2}-(z+\chi+\mu_{2})S_{2}$$

$$\frac{dE_{2}}{dt}={(\Lambda}_{2}^{m}+\Lambda_{2}^{c})S_{2}+gE_{3}-(z+\chi+\eta+\mu_{2})E_{2}$$

$$\frac{dI_{2}}{dt}=\eta E_{2}+\sigma T_{2}+gI_{3}-(z+\chi+\omega+\delta+\mu_{2})I_{2}$$

$$\frac{dT_{2}}{dt}=\omega I_{2}+gT_{3}-(z+\chi+\sigma+\varphi\delta+\mu_{2})T_{2}$$

Client population

$$\frac{dS_{3}}{dt}=zS_{2}-{(\Lambda}_{3}^{m}+\Lambda_{3}^{c}+\Lambda_{3}^{co})S_{3}-(g+\mu_{3})S_{3}$$

$$\frac{dE_{3}}{dt}={(\Lambda}_{3}^{m}+\Lambda_{3}^{c}+\Lambda_{3}^{co})S_{3}+zE_{2}-(g+\eta+\mu_{3})E_{3}$$

$$\frac{dI_{3}}{dt}=\eta E_{3}+\sigma T_{3}+zI_{2}-(g+\omega+\delta+\mu_{3})I_{3}$$

$$\frac{dT_{3}}{dt}=\omega I_{3}+zT_{2}-(g+\sigma+\varphi\delta+\mu_{3})T_{3}$$

FSW population

$$\frac{dS_{4}}{dt}=\kappa S_{1}-{(\Lambda}_{4}^{m}+\Lambda_{4}^{c}+\Lambda_{4}^{co})S_{4}-(\gamma+\mu_{4})S_{4}$$

$$\frac{dE_{4}}{dt}={(\Lambda}_{4}^{m}+\Lambda_{4}^{c}+\Lambda_{4}^{co})S_{4}+\kappa E_{1}-(\gamma+\eta+\mu_{4})E_{4}$$

$$\frac{dI_{4}}{dt}=\eta E_{4}+\sigma T_{4}+\kappa I_{1}-(\gamma+\omega+\delta+\mu_{4})I_{4}$$

$$\frac{dT_{4}}{dt}=\omega I_{4}+\kappa T_{1}-(\gamma+\sigma+\varphi\delta+\mu_{4})T_{4}$$

MSM population-young

$$\frac{dS_{5}}{dt}=\chi S_{2}-{(\Lambda}_{5}^{m}+\Lambda_{5}^{c}+\Lambda_{5*}^{m}+\Lambda_{5*}^{c})S_{5}-(\zeta+\mu_{5})S_{5}$$

$$\frac{dE_{5}}{dt}={(\Lambda}_{5}^{m}+\Lambda_{5}^{c}+\Lambda_{5*}^{m}+\Lambda_{5*}^{c})S_{5}+\chi E_{2}-(\zeta+\eta+\mu_{5})E_{5}$$

$$\frac{dI_{5}}{dt}=\eta E_{5}+\sigma T_{5}+\chi I_{2}-(\zeta+\omega+\delta+\mu_{5})I_{5}$$

$$\frac{dT_{5}}{dt}=\omega I_{5}+\chi T_{2}-(\zeta+\sigma+\varphi\delta+\mu_{5})T_{5}$$

MSM population-old

$$\frac{dS_{6}}{dt}=\zeta S_{5}-{(\Lambda}_{6}^{m}+\Lambda_{6}^{c}+\Lambda_{6*}^{m}+\Lambda_{6*}^{c})S_{6}-\mu_{6}S_{6}$$

$$\frac{dE_{6}}{dt}={(\Lambda}_{6}^{m}+\Lambda_{6}^{c}+\Lambda_{6*}^{m}+\Lambda_{6*}^{c})S_{6}+\zeta E_{5}-(\eta+\mu_{6})E_{6}$$

$$\frac{dI_{6}}{dt}=\eta E_{6}+\sigma T_{6}+\zeta I_{5}-(\omega+\delta+\mu_{6})I_{6}$$

$$\frac{dT_{6}}{dt}=\omega I_{6}+\zeta T_{5}-(\sigma+\varphi\delta+\mu_{6})T_{6}$$

**Model parameterisation and calibration**

Recent model parameter and calibration data for FSW, clients and MSM were obtained from three integrated behavioural and biological assessment (IBBA) surveys undertaken in Dakar, Senegal, from 2015-2016^[1]^ (IBBA client data are unpublished, 2016). Specifically, behavioural data for FSW, clients and MSM on the current frequency of commercial sex, frequency of main and casual partners, and associated frequency of sex and condom use for these different partnership types came from these IBBA surveys.

In addition, older published and unpublished IBBA surveys were used to determine whether risk behaviour has changed over time, and how the HIV epidemic in different risk groups has evolved. Importantly, this included past trends in the consistency of condom use for the different risk groups, as described below. Unfortunately, as surveys generally did not use the same measures for behavioural quantities, it was difficult to precisely evaluate how specific behaviours changed over time, and thus uncertainty was incorporated into those trends. A summary of all the bio-behavioural surveys used in the modelling is included in Supplementary Table S1; model parameterisation from 2015-2016 surveys is summarised in Table 1, with the full parameter table given in supplementary table S2.

Adult population HIV-related epidemiological and sexual behaviour data was obtained from DHS for 2005 and 2010^[2, 3]^. Condom use amongst low-risk partnerships was low in these surveys (1.4-4.2% with main partners during last act^[3]^). The modelled condom use trends for FSW and MSM are discussed below in detail and are shown in Figure S1, with the modelled trends for clients assumed to be the same as for FSW, and general population assumed to be constant for main partners and similar to FSW for casual partners.

Senegal population size estimates for 1980 to 2020 and gender specific death rates were obtained from UNDP.^[4]^ Population growth rates were fit to give the increase in national population size over this period, which was then factored down to the population size of Dakar. Population size estimates for FSW and MSM were produced as part of the 2015 IBBA surveys using the service multiplier and unique object methods^[5]^. The population size of clients was estimated through balancing the overall demand for commercial sex of FSW with that of clients, as described in the supplementary materials.

ART coverage data for the overall Senegal population over time came from the World Bank^[6, 7]^ (Supplementary figure S2), which suggested ART recruitment increased from negligible levels in 2000 up to 44% coverage of HIV-infected individuals (43% (35-50%) and 49% (36-60%) among males and females respectively) in 2015. The same coverage trends for males were assumed for clients because of a lack of data, and for MSM because they were consistent with data from the 2015 IBBA on the proportion of HIV-infected MSM that were virally suppressed (assuming 75% viral suppression amongst those on ART in Dakar)^[8]^. For FSW, the World Bank ART coverage trends were scaled up because data from the 2016 IBBA found 74% of HIV-infected FSW were virally suppressed, suggesting higher ART coverage levels of 93% if 75% were virally suppressed^[8]^. ART recruitment rates were varied to give the increase in ART coverage between 2000 and 2015 for each group. Other HIV biological parameters were obtained from literature (Supplementary Table 2).

Uncertainty distributions were assigned to all model parameters except the transition rates to the FSW, client, and MSM groups, which were varied to fit the corresponding population sizes for each group. All uncertainty distributions were assumed to be uniform except for the proportion of females that are FSW (0.28-1.2%) and proportion of men that are MSM (0.76-2.4%), which were sampled from a triangular distribution because the mean of the proportions and range were skewed. Most parameters were fixed over time except for the rate of ART recruitment and levels of condom use, as already described. The only additional time varying parameter was the frequency of sex for MSM with their main and casual male partners, which data suggest increased between 2007^[9]^ to 2016^[1]^. The model assumed this behaviour was constant up to 2007^[10, 11]^ and increased linearly up to a higher constant value between 2007 to 2016.

To incorporate uncertainty, 10,000 parameter sets were randomly sampled from parameter ranges given in Supplementary Table 2 (summarised in Table 1 in main text). For each sampled parameter set, the transition rates into FSW and MSM were firstly calibrated to give the steady sampled FSW and MSM proportion. Similarly, the proportion of men that are clients was estimated by balancing the commercial sex acts reported by FSW and clients, and the transition rate into the client population was calibrated to give this steady client proportion. Using these estimated transition rates, the full model was then run to endemic level by seeding a 1-2% HIV prevalence amongst FSW, a third of that in the client population and 1-2% HIV prevalence in the MSM population. These runs assumed no increase in condom use or ART coverage over time, and the obtained endemic HIV prevalences were then used to rerun the model while incorporating the function that attempts to capture the effect of heterogeneity in behavioural risk (see model description above). For these new runs, we also allow condom use and the frequency of sex for male partners of MSM to vary over time and calibrate the ART recruitment rates to give the increases in ART coverage described above. Any runs producing HIV prevalence projections that agreed with early IBBA HIV prevalence data for FSW (1990 or 1995) and clients (1999) and recent HIV prevalence data for young MSM from 2014 to 2016 were selected as a model fit. Based on numerous data estimates for FSW, we assumed a range for the HIV prevalence amongst FSW of 2.0-10.0% in 1990 and 5.0-15.0% in 1995, 1.3-5.1% in clients (1999) and 9.7-37.7% in MSM (2014-2016), with these prevalence ranges being shown in Figure 3, with their data sources included in supplementary Table 1. The wide range for MSM is due to contrasting estimates from two IBBA surveys in 2014 and 2016. Other HIV prevalence data from KP, and for the overall population from 2005 and 2010 (from DHS surveys), are also shown in Figure 3, but were not fit to; instead they were used to validate the realism of the model projections.

**Condom use assumptions**

*Condom use* for *FSW and clients*

Early on (1985-1990), FSW studies (including client data) carried out in Dakar and other cities in Senegal^[12-15]^ suggest condom use for commercial sex ranged between about 50-90% although this is uncertain due to the differences in measures used. Over a similar period (1988-1997), condom distribution data suggests a 10-fold increase in the availability of condoms from 800,000 in 1988 to 7 million in 1997^[16]^. This resulted in 99% of FSWs reporting easy access to condoms in 1997. For the period 1997-2004, FSW IBBA studies suggest condom use was high, covering about 84-98% in last act^[17-19]^, similar to what was reported in the 2016 IBBA FSW survey^[1]^. Importantly, though, the recent client IBBA survey from 2016 suggests that condom use was 54-64% in last sex acts with commercial partners (client IBBA unpublished 2016), much lower than reported in standard FSW surveys. This agrees with a recent FSW survey from Dakar that used novel methods to reduce reporting bias, which also found a lower level of condom use of 78% in last commercial sex acts (Aurelia Lepine, personal communication 2016).

Based on this data, we modelled condom use in commercial sex for vaginal intercourse (VI) starting at a low (0-20%) level in 1985, starting to increase between 1985 and 1993 (because of uncertainty in early measure) to a moderate to high level by 1998 that plateaus at 54-90% based on the client and FSW 2016 survey (Figure 2a, main text). Condom use for anal intercourse (AI) with commercial partners is assumed to be half that of VI for all years, based on comparing VI and AI levels of condom use from the 2016 IBBA FSW and client surveys.

Data suggests condom use for regular partners of FSWs was low (18.2% always) in 2000^[19]^, and moderate (62% always^[20]^ or 60% in last sex act^[1]^) in 2003 and 2016. We therefore assumed that condom use for VI with main partners is 0% in 1985, (10-20%) in 1998 and increased to 37-60% by 2003, and stable thereafter (lower bound of 37% is assumed because of likely bias in condom use reporting as found for commercial sex) (Figure 2b, main text). Based on comparing condom use for main and casual partners in the 2016 FSW IBBA, casual partner VI condom use was assumed to be 1-1.5 times higher than for main partners for all years. Additionally, also based on the 2016 FSW IBBA, AI condom use with main and casual partners was assumed to be 0.6-1 times lower than for VI condom use with main and casual partners, respectively.

*Condom use for MSM*

In 2001, an assessment to understand the lives of MSM found very low condom use (14-23% in last act) for sex with men but higher (37% in last act) with women^[21]^. Condom use in sex with men was higher (55-58% inconsistent condom use) in a 2004 survey,^[10]^ which is probably similar to what was found (73 and 82% condom use in last act) in surveys from 2003 and 2005^[11]^, as well as surveys from 2007 (76.4% in last sex act)^[22]^ and 2016 (81-84% in last sex act - 2016 IBBA). Based on these trends, we assumed there was no condom use among MSM with male main and casual partners in 1985, low (10-30%) in 2001, then 70-85% by 2003-2007 and constant thereafter (Figure 2c, main text). In the 2016 IBBA, condom use with regular and casual male partners was similar, so we assumed the same condom use for all male partnerships. With female main and casual partners, condom use for VI (assumed same for AI based on 2016 MSM IBBA) was assumed to be zero in 1985, 20-37% in 2001, increasing to 50-65% by 2004 and to a maximum of and 67-95% in 2016 based on data from the same surveys as for condom use for sex between MSM (Figure 2(d)). We assume all increases in condom use are linear and to incorporate bias in reporting all rates were multiplied by 0.7-1.0 (lower bound is chosen to give overall lower bound of 50%).

**ANCOVA analysis**

Analysis of covariance showed that most of the variability in the 10-year PAF for sex between men in 2015 is due to uncertainty in HIV prevalence among clients (7.7% of variability), with factor for linear increase in frequency of sex among MSM (5.3%) and low risk female frequency of main partners (5.2%) also playing a role (Figure S3A). Uncertainty in young MSM frequency of main female partners (14.3%) also contribute most to the variability in the 10-year PAF for commercial sex, with bias factor for MSM condom use with partners (7.6%), and HIV prevalence among clients (6.2%) (Figure S3B) also being important.

**Tables**

**Supplementary Table 1**. Details of surveys used to parameterise and calibrate the model for different risk groups in Dakar.

| Risk group | Year  of survey | How sampled | Settings sampled | Dakar Sample size | HIV-1 prevalence  % (95% CI) | Reference |
| --- | --- | --- | --- | --- | --- | --- |
| Clients | 1989-1991 | Male outpatients seeking treatment at an STD centre were recruited | Dakar | 975 | - | ^[15]^ |
|  | 1999 | For 7 brothels, successive males entering to buy sex sampled during randomly selected time slot. | Dakar | 1083 | 3.2 (1.1-5.5)* | ^[23]^ |
|  | 2016 | Respondent driven sampling | Dakar | 602 | 1.2 (0.5-2.1) | Unpublished Client IBBA 2016 undertaken by project team |
| MSM | 2000-1 | Snowball sampling using recognised leaders to recruit other men | Dakar | 250 | NA | ^[21]^ |
|  | 2001 | Snowball sampling | Dakar | 250 | NA | ^[11]^ |
|  | 2003/5 | Snowball sampling | Dakar | 258/290 | NA | ^[11]^ |
|  | 2004 | Snowball referrals with 24 initial peer leaders recruited through informal networks where MSM meet | Dakar, Thies, Mbour, Kaolack, Saint Louis | 297 | 20.7 (16.2-26.0)* | ^[10]^ |
|  | 2007 |  | Dakar, Thies, Mbour, Saint Louis | 306 | 20.2 (16.8-23.7)* | ^[9][22]^ |
|  | 2016 | Respondent driven sampling | Dakar | 727 | Young MSM: 28.6 (19.6-37.7)  Old MSM: 37.0  (14.3-59.8) | ^[1]^ MSM IBBA 2016 |
| FSW | 1985-90 | Recruited from registered FSW visiting clinic for annual health evaluation | Dakar, Ziguinchor, Kaolack | 1275 | NA | ^[12]^ |
|  | 1990 | Open cohort of registered FSWs whose samples were obtained from clinic visits -Longitudinal study 1985-2004 | Dakar | 3910 | 3.0 (2-10) | ^[24]^ |
|  | 1990-1993 | FSW presenting to STD clinics | Dakar | 975 | NA | ^[15]^ |
|  | 1990 | Registered FSW attending special clinic for screening examination | Dakar | 374 | NA | ^[13]^ |
|  | 1995 | Same as above |  |  | 6.3 (5.0-15.0) | ^[24]^ |
|  | 2000 | One stage cluster sampling | Dakar | 390 | 6.0 (2.8-9.1) | ^[18]^ |
|  | 2000-2004 | FSWs attending STD clinics | Dakar, Mbour, Sebikotane | 623 | - | ^[19]^ |
|  | 2006 | Two stage cluster sampling | Multi-city | NA | 8.7 (6.0-12.0)* | ^[25]^ |
|  | 2010 | Two stage cluster sampling | Multi-city | NA | 10.1 (7.0-14.0)* | ^[26]^ |
|  | 2015 | Two stage cluster sampling | Multi-city | NA | 4.1 (2.0-7.0)* | ^[27]^ |
|  | 2016 | RDS |  | 758 | 6.6 (4.6-9.1) | ^[1]^ FSW IBBA 2016 |
|  |  |  |  |  |  |  |
| Overall female | 2005 | Two-stage stratified random samples of households | National | 1233 | 0.62 (0.30-1.2)* | ^[2]^ |
|  | 2010 |  | National | 1379 | 0.30 (0.12-0.71)* | ^[3]^ |
| Overall male | 2005 | Two-stage stratified random samples of households | National | 1047 | 0.40 (0.13-0.88)* | ^[2]^ |
|  | 2010 |  | National | 1285 | 0.40 (0.14-0.80)* | ^[3]^ |

*Adjusted to give HIV-1 prevalence in Dakar – prevalence only given across sites and/or just HIV-1 and HIV-2 combined

NA – data not available

**Supplementary Table 2(a).** Demographic, sexual and behavioural parameters of female sex workers, clients, MSM and low risk populations.

| **Types of model input** | **Definition of model input** | **Male** | **Female** | **References and comments** |
| --- | --- | --- | --- | --- |
| **Population size & demographic inputs** | | | | |
|  | Initial size of sexually active population (15-49 years old) | 885,570 | 895,266 | Dakar census data^[28]^ |
|  | Life expectancy in years | 51.3 | 54.0 | The number of remaining years of life expected to live by individuals at age 15 for Senegal^[4]^ |
| **FSW and client population and sexual behaviour** | | | | |
| **Proportion of adults that are clients or FSW** | Proportion of adult females and males that are FSW or clients | Varied to balance commercial sex acts | 0.47% (0.3-0.9%) | Size estimates obtained in 2016 by using the service multiplier and unique object methods^[5]^ |
| **Commercial partners of clients and FSW** | Average frequency of FSWs per client and clients per FSW per year | 62.4-78.0 | 368.7-691.2 | FSW IBBA 2016, Clients IBBA 2016, with data being expanded due to data from earlier surveys.^[14, 29]^ No suggestion of change over time |
|  | Percentage of commercial sex acts that are anal | 3.8-6.5% | 2.1-6.5% | FSW IBBA 2016 |
|  | Condom use per vaginal sex act with commercial partner | Varied (time dependent) | Varied (time dependent) | See section above and Figure 2 |
|  | Condom use per anal sex act with commercial partners | Varied (time dependent) | Varied (time dependent) | See section above |
| **Main partners of clients and FSW** | Average frequency of main partners per year | 1.3-2.6 | 0.5-0.7 | FSW IBBA 2016 and Client IBBA 2016. For clients, main partners of clients that are FSW have been accounted for. |
|  | Frequency of vaginal sex with main partners per year | 73.6-124.6 | 78.5-117.5 | FSW IBBA 2016, Client IBBA 2016 |
|  | Frequency of anal sex with main partners per year | 3.4-11.1 | 3.6-15.6 | Used estimates from FSW IBBA 2016 and Client IBBA 2016 to create a combined range that we sample from |
|  | Condom use per vaginal sex act with main partners | Varied (time dependent) | Varied (time dependent) | See section above |
|  | Condom use per anal sex act with casual partners | Varied (time dependent) | Varied (time dependent) | See section above |
| **Casual partners of clients and FSW** | Average frequency of casual partners per year | 0.2-1.4 | 0.5-1.6 | Client and FSW IBBA 2016, but removed proportion with FSWs in client estimate |
|  | Frequency of vaginal sex with casual partners per month | 2.1-5.9 | 4.2-8.2 | FSW IBBA 2016, Client IBBA 2016 |
|  | Frequency of anal sex with casual partners per month | 0.1-0.8 | 0.1-1.2 | FSW IBBA 2016, Client IBBA 2016 |
|  | Condom use per vaginal sex act with casual partner | Varied (time dependent) | Varied (time dependent) | See section above |
|  | Condom use per anal sex act with casual partner | Varied (time dependent) | Varied (time dependent) | See section above |
|  |  |  |  |  |
| **Duration of sex work** | Average duration of sex work in years | 12-20 | 2-14 | Average duration of current FSW is 7.2 (6.8-7.7) or IQR of 3-10, whereas for clients it is 13.3 (12.4-14.3) years (FSW IBBA 2016, Client IBBA 2016). Extend duration to 14 years for FSW and 20 for clients to give estimate of ever duration, and shorten lower bound for FSWs because earlier data^[14, 18, 19]^ suggests could be shorter (2 years) |
| **MSM population and sexual behaviour** | | **Male partners** | **Female partners** | MSM have male and female main and casual partners |
| MSM population size | Proportion of adult (15-49 years) males that are MSM | 1.2% (0.8-2.3%) |  | Size estimates obtained in 2016 by using the service multiplier and unique object methods^[5]^ |
| Main partners of young MSM | Frequency of main partner per year | 0.6-0.8 | 0.4-1.5 | MSM IBBA 2016 |
|  | Frequency of anal sex with main partner per year (evenly split between receptive and anal with male partner) | 91.0-120.6 | 1.6-9.5 | MSM IBBA 2016 – For male partners it is sum of frequency of insertive and receptive sex in last week. |
|  | Frequency of vaginal sex with main partner per year | - | 56.2-74.4 | MSM IBBA 2016 |
|  | Condom use for anal sex with main partner | Varied (time dependent) | Varied (time dependent) | See section above |
|  | Condom use for vaginal sex with main partner | - | Varied (time dependent) | See section above |
| Casual partners of young MSM | Frequency of casual partners per year | 2.5-9.3 | 0.55-5.1 | MSM IBBA 2016 |
|  | Frequency of anal sex with casual partner per month (evenly split between receptive and insertive with male partner) | 6.7-8.2 | 0.04-1.0 | MSM IBBA 2016 |
|  | Frequency of vaginal sex with casual female partner per month | - | 2.6-6.4 | MSM IBBA 2016 |
|  | Condom use for anal sex with casual partner | Varied (time dependent) | Varied (time dependent) | See section above |
|  | Condom use for vaginal sex with casual partner |  | Varied (time dependent) | No sufficient data for female partner so assume same as for main female partner |
|  | Average duration in young MSM group | 10-15 years |  | MSM IBBA 2016 found average age started was 17.7 years (16.8-18.6) and very few over 30 years so say leave after 10-15 years |
| **Differences for old MSM^a^ (>=30 years)** | |  |  |  |
| Casual partners of old MSM | Frequency of casual partners per year | 1.3-9.3 | 0.14-5.1 | Male partners are 0.5-1 times that for young MSM and female partners are 0.25-1 times that for young MSM - MSM IBBA 2016 |
|  | Frequency of anal sex for casual male partners per year (evenly split between receptive and insertive) | 13.1-17.4 | - | Frequency is twice that for young MSM - MSM IBBA 2016 |
|  | Condom use for anal sex with main male partner | Varied (time dependent) | - | (0.75-1) times that for young MSM - MSM IBBA 2016 |
|  | Condom use for anal sex with casual male partner | Varied (time dependent) | - | (0.65-1) times that for young MSM - MSM IBBA 2016 |
| **Low risk female and male** | | | | |
| Main partners for low risk | Proportion with current main partner | 56-66% | 56-66% | DHS 2010 |
|  | Frequency of sex with main partners per year | 31.6-61.8 | 31.6-61.8 | DHS 2010 – assume same for male and female |
|  | Condom use with main partner | 1.4-4.2 | 1.4-4.2 | DHS 2010 – assume constant over time and same for males and females |
| Casual partners for low risk | Proportion that had casual partners in last year | 12.3-14.2% | 12.3-14.2% | DHS 2010 – percentage that have >1 partners in last year. Females is likely under-estimated and so assumed same as males report |
| Casual partners for low risk | Frequency of casual partners in last year | 0.15-0.26 | 0.12-0.15 | DHS 2010 – Males and females that have >1 partner in last year report 2.47 (2.23-2.85) and 2.03 (2.00-2.07) partners in last year, respectively. Remove 1 for main partners to have number of casual partners and multiply by proportion that have casual partners (12.3-14.2%, as males report) |
|  | Frequency of sex for casual in last 12 months | 6.3 (5.6-7.2) | 12.8 (10.9-14.7) | DHS 2010 when last recent partner is casual/girlfriend/relative |
|  | Condom use with casual partner | Varied (Time dependent) | Varied (Time dependent) | DHS 2010 showed same condom use as that of FSW with casual partners. Assume same condom use trend as for FSW |

**^a^**Parameters here are different for young and old MSM. All other parameters for old MSM are the same as for young MSM.

**Supplementary Table 2(b).** HIV epidemiological parameters

| **Parameter definition** | **Range** | **References** |
| --- | --- | --- |
| Duration of acute HIV stage in years | 0.24 (0.10-0.38) | ^[30]^ |
| Duration of pre-AIDS stage in years | 0.75 (0.40-1.1) | ^[30]^ |
| Relative infectiousness of those in the acute stage of HIV infection compared with the chronic stage | 11.7 (4.5-18.8) | ^[31]^ |
| HIV related death rate per year | 0.1 |  |
| ART recruitment rate | Varied to fit coverage |  |
| Loss to follow up from ART per year | 3% (2-4%) | ^[32]^ |
| Death rate while of ART per year | 2.5-4.5% | ^[33-36]^ |
| Per-sex-act (%) efficacy of ART in reducing HIV transmission risk in anal or vaginal sex for those that are virally suppressed. | 85-96% amongst those virally suppressed with 75% of people on ART assumed to be virally suppressed | ^[37]^  ^[8, 38]^ |
| Per-sex-act (%) efficacy of condoms in reducing HIV transmission risk in vaginal sex | 80 (66-94) | ^[39]^ |
| Per-sex-act (%) efficacy of condoms in reducing HIV transmission risk in anal sex | 80 (66-94) | ^[40]^ |
| Relative risk of acquiring HIV from receptive AI vs. receptive VI | 2.0-18.0 | ^[41]^ |
| Relative risk of acquiring HIV from insertive AI vs. insertive VI | 1.0-2.0 | ^[41]^ |
| Risk of HIV infection per receptive vaginal intercourse with an infected partner | 0.0033 (0.0006-0.006) | ^[31, 39]^ |
| Risk of infection per insertive vaginal intercourse with an infected partner | 0.0033 (0.0006-0.006) | ^[31]^Assume same as receptive VI |
| Efficacy (%) of circumcision for reducing susceptibility of males | 54% (38-66%) | ^[42]^ |

**Figures**


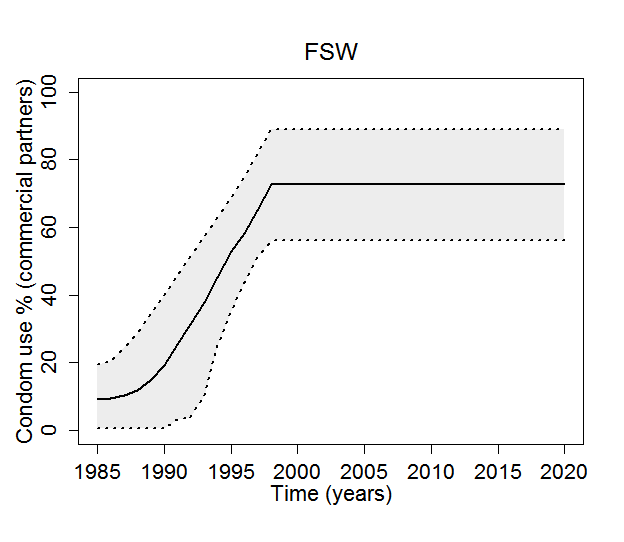


**Figure S1**(a). FSW condom use with commercial partners for vaginal intercourse. Assume condom use for anal intercourse is half that of vaginal intercourse for all the years.


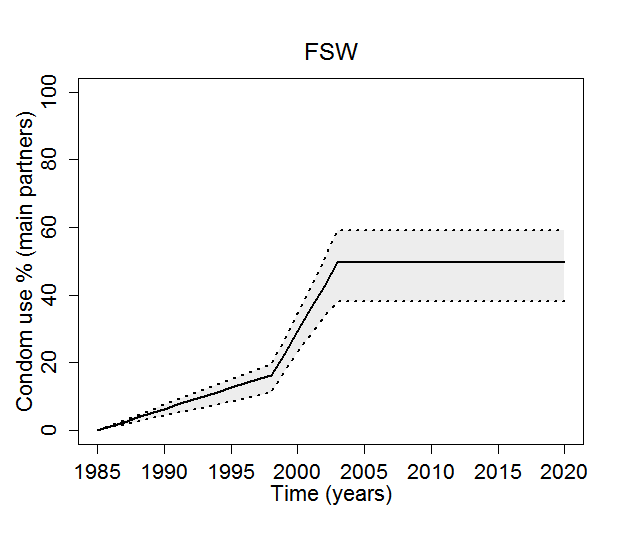


**Figure S1**(b). FSW condom use with main partners for vaginal intercourse (VI). Condom use for anal intercourse (AI) with main partners is assumed to be 0.6-1.0 times that for VI. FSW condom use with casual partners for VI is 1-1.5 times that for main partners VI; AI with casual partners is 0.6-1.0 times that for VI with casual partners.


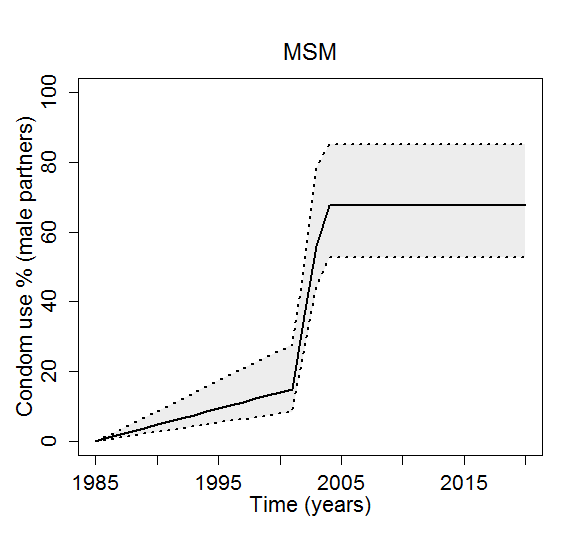


**Figure S1(c).** MSM condom use with male regular and casual partners. Assume some bias in reporting so all rates are multiplied by a bias factor of 0.7-1.0 – lower bound of 0.7 chosen to give overall lower bound of 0.5.


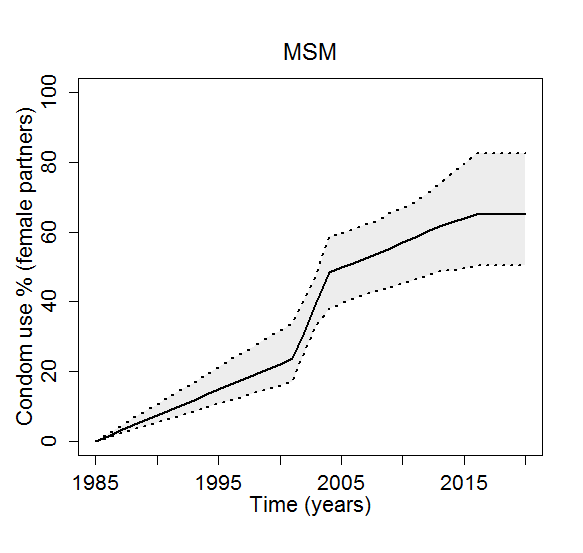


**Figure S1(d).** MSM condom use with female main and casual partners. Condom use is assumed to be the same for VI and AI.

**Figure S1**. Modelled condom use trends for female sex workers (FSW) and men who have sex with men (MSM)

**Figure S2.** ART coverage in Senegal from 2000 from UNAIDS AIDS info^[7]^ and World Bank^[6]^


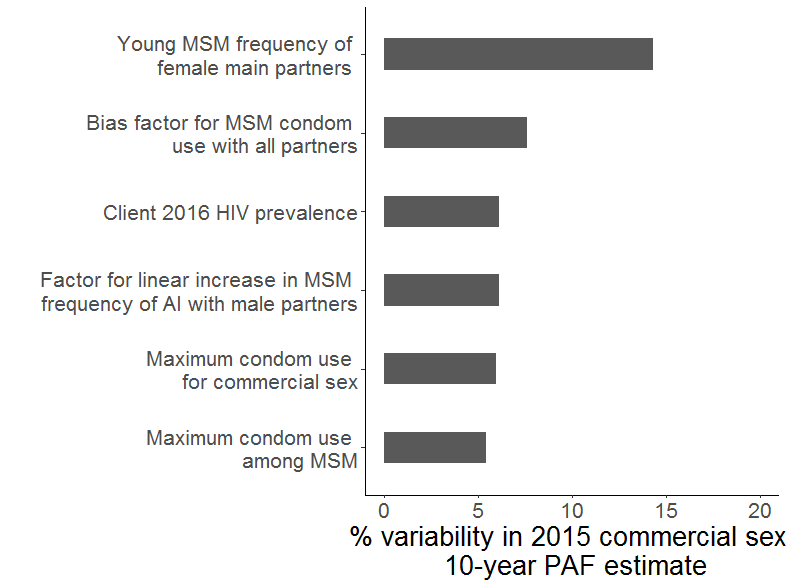


(A)


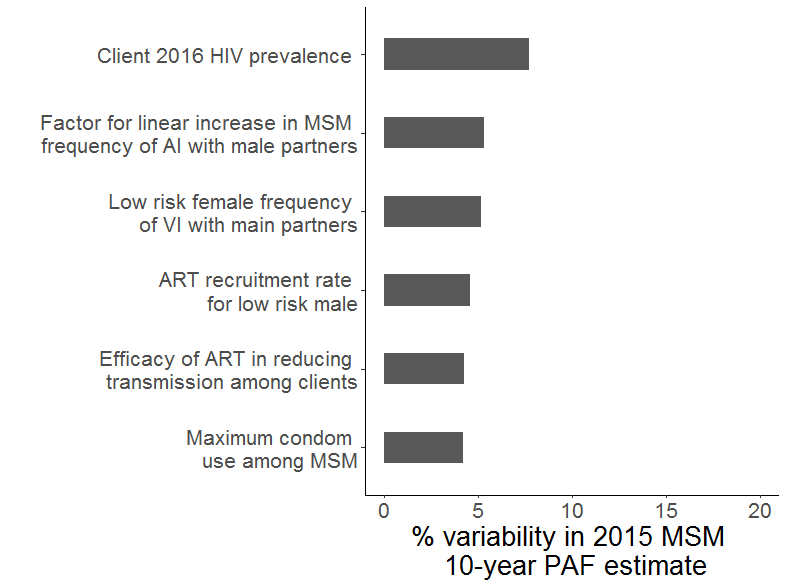


(B)

**Figure S3**. Analysis of covariance results of parameters that contribute more than 4% variability to the 2015 (A) 10-year commercial sex PAF and (B) 10-year MSM PAF.

**References**

1. Lyons CE, Ketende S, Diouf D, Drame FM, Liestman B, Coly K, et al. **Potential Impact of Integrated Stigma Mitigation Interventions in Improving HIV/AIDS Service Delivery and Uptake for Key Populations in Senegal**. *J Acquir Immune Defic Syndr* 2017; 74 Suppl 1:S52-S59.

2. DHS. **Enquête Démographique et de Santé Sénégal 2005**. In; 2005.

3. DHS-MICS. **2010-11 Demographic and Health Survey and Multiple Indicator Cluster Survey**. In; 2010.

4. UNPD. **United Nations, Department of Economic and Social Affairs, Population Division. United Nations World Population Prospects: The 2015 Revision.** <https://esa.un.org/unpd/wpp/>**.** . In. 2015 ed; 2015.

5. Paz-Bailey G, Jacobson JO, Guardado ME, Hernandez FM, Nieto AI, Estrada M, et al. **How many men who have sex with men and female sex workers live in El Salvador? Using respondent-driven sampling and capture-recapture to estimate population sizes**. *Sex Transm Infect* 2011; 87(4):279-282.

6. World-Bank. **The Wolrd Bank Data, Antiretroviral therapy coverage (% of people living with HIV)**. In; 2016.

7. UNAIDS-AIDSinfo. **Available at** <http://aidsinfo.unaids.org/>. In; 2016.

8. Diouara AA, Ndiaye HD, Guindo I, Bangoura N, Cisse M, Edmond T, et al. **Antiretroviral treatment outcome in HIV-1-infected patients routinely followed up in capital cities and remote areas of Senegal, Mali and Guinea-Conakry**. *J Int AIDS Soc* 2014; 17:19315.

9. Wade AS, Larmarange J, Diop AK, Diop O, Gueye K, Marra A, et al. **Reduction in risk-taking behaviors among MSM in Senegal between 2004 and 2007 and prevalence of HIV and other STIs. ELIHoS Project, ANRS 12139**. *AIDS Care* 2010; 22(4):409-414.

10. Wade AS, Kane CT, Diallo PA, Diop AK, Gueye K, Mboup S, et al. **HIV infection and sexually transmitted infections among men who have sex with men in Senegal**. *AIDS* 2005; 19(18):2133-2140.

11. Geibel S, Tun W, Tapsoba P, Kellerman S. **HIV vulnerability of men who have sex with men in developing countries: Horizons studies, 2001-2008**. *Public Health Rep* 2010; 125(2):316-324.

12. Kanki P, M'Boup S, Marlink R, Travers K, Hsieh CC, Gueye A, et al. **Prevalence and risk determinants of human immunodeficiency virus type 2 (HIV-2) and human immunodeficiency virus type 1 (HIV-1) in west African female prostitutes**. *Am J Epidemiol* 1992; 136(7):895-907.

13. Ndoye I, Mboup S, De Schryver A, Van Dyck E, Moran J, Samb ND, et al. **Diagnosis of sexually transmitted infections in female prostitutes in Dakar, Senegal**. *Sex Transm Infect* 1998; 74 Suppl 1:S112-117.

14. Langley CL, Benga-De E, Critchlow CW, Ndoye I, Mbengue-Ly MD, Kuypers J, et al. **HIV-1, HIV-2, human papillomavirus infection and cervical neoplasia in high-risk African women**. *AIDS* 1996; 10(4):413-417.

15. Thior I, Diouf G, Diaw IK, Sarr AD, Hsieh CC, Ndoye I, et al. **Sexually transmitted diseases and risk of HIV infection in men attending a sexually transmitted diseases clinic in Dakar, Senegal**. *Afr J Reprod Health* 1997; 1(2):26-35.

16. UNAIDS. **HIV prevention needs and successes: a tale of three countries**. In. Geneva: UNAIDS; 2001.

17. Meda N, Ndoye I, M'Boup S, Wade A, Ndiaye S, Niang C, et al. **Low and stable HIV infection rates in Senegal: natural course of the epidemic or evidence for success of prevention?** *AIDS* 1999; 13(11):1397-1405.

18. Laurent C, Seck K, Coumba N, Kane T, Samb N, Wade A, et al. **Prevalence of HIV and other sexually transmitted infections, and risk behaviours in unregistered sex workers in Dakar, Senegal**. *AIDS* 2003; 17(12):1811-1816.

19. Wang C, Hawes SE, Gaye A, Sow PS, Ndoye I, Manhart LE, et al. **HIV prevalence, previous HIV testing, and condom use with clients and regular partners among Senegalese commercial sex workers**. *Sex Transm Infect* 2007; 83(7):534-540.

20. Homaifar N, Wasik SZ. **Interviews with senegalese commercial sex trade workers and implications for social programming**. *Health Care Women Int* 2005; 26(2):118-133.

21. Niang CK, Tapsoba P, Weiss E, Diagne M, Niang Y, Moreau MA, et al. **'It's raining stones': stigma, violence and HIV vulnerability among men who have sex with men in Dakar, Senegal**. *Culture, Health & Sexuality* 2003; 5(6):499-512.

22. Larmarange J, Wade AS, Diop AK, Diop O, Gueye K, Marra A, et al. **Men who have sex with men (MSM) and factors associated with not using a condom at last sexual intercourse with a man and with a woman in Senegal**. *PLoS One* 2010; 5(10).

23. Gomes Do Espirito Santo ME, Etheredge GD. **How to reach clients of female sex workers: a survey ‘‘by surprise’’ in brothels in Dakar, Senegal**. *Bull World Health Organ* 2002; 80:709-713.

24. Hamel DJ, Sankale JL, Eisen G, Meloni ST, Mullins C, Gueye-Ndiaye A, et al. **Twenty years of prospective molecular epidemiology in Senegal: changes in HIV diversity**. *AIDS Res Hum Retroviruses* 2007; 23(10):1189-1196.

25. ENSC. **ENQUÊTE NATIONALE DE SURVEILLANCE COMBINEE DES IST ET DU VIH/SIDA, ENSC 2006**. In; 2006.

26. ENSC. **ENQUÊTE NATIONALE DE SURVEILLANCE COMBINEE DES ISTET DU VIH/SIDA, ENSC 2010**. In; 2010.

27. ENSC. **ENQUÊTE NATIONALE DE SURVEILLANCE COMBINEE DES IST ET DU VIH/SIDA, ENSC 2015**. In; 2015.

28. ANSD. **Agence Nationale de la Statistique et de la Démographie: RAPPORT DEFINITIF RGPHAE 2013**. In; 2013. pp. 52-70.

29. Donnelly C, Leisenring W, Kanki P, Awerbuch T, Sandberg S. **Comparison of transmission rates of HIV-1 and HIV-2 in a cohort of prostitutes in Senegal**. *Bull Math Biol* 1993; 55(4):731-743.

30. Hollingsworth TD, Anderson RM, Fraser C. **HIV-1 transmission, by stage of infection**. *J Infect Dis* 2008; 198(5):687-693.

31. Boily MC, Baggaley RF, Wang L, Masse B, White RG, Hayes RJ, et al. **Heterosexual risk of HIV-1 infection per sexual act: systematic review and meta-analysis of observational studies**. *Lancet Infect Dis* 2009; 9(2):118-129.

32. Gabillard D, Lewden C, Ndoye I, Moh R, Segeral O, Tonwe-Gold B, et al. **Mortality, AIDS-morbidity, and loss to follow-up by current CD4 cell count among HIV-1-infected adults receiving antiretroviral therapy in Africa and Asia: data from the ANRS 12222 collaboration**. *J Acquir Immune Defic Syndr* 2013; 62(5):555-561.

33. Etard JF, Ndiaye I, Thierry-Mieg M, Gueye NF, Gueye PM, Laniece I, et al. **Mortality and causes of death in adults receiving highly active antiretroviral therapy in Senegal: a 7-year cohort study**. *AIDS* 2006; 20(8):1181-1189.

34. De Beaudrap P, Diouf A, Bousso Niang K, groupe d'etude de la Cohorte A. **[Clinical and biological effectiveness of antiretroviral therapy in the ANRS 1215 cohort]**. *Bull Soc Pathol Exot* 2014; 107(4):230-233.

35. Bastard M, Fall MB, Laniece I, Taverne B, Desclaux A, Ecochard R, et al. **Revisiting long-term adherence to highly active antiretroviral therapy in Senegal using latent class analysis**. *J Acquir Immune Defic Syndr* 2011; 57(1):55-61.

36. Laurent C, Tchatchueng Mbougua JB, Ngom Gueye NF, Etard JF, Diouf A, Landman R, et al. **Long-term effectiveness and safety of didanosine combined with lamivudine and efavirenz or nevirapine in antiretroviral-naive patients: a 9-year cohort study in Senegal**. *Trop Med Int Health* 2011; 16(2):217-222.

37. Cohen MS, Chen YQ, McCauley M, Gamble T, Hosseinipour MC, Kumarasamy N, et al. **Prevention of HIV-1 infection with early antiretroviral therapy**. *N Engl J Med* 2011; 365(6):493-505.

38. Diouara AA, Diop-Ndiaye H, Kebe-Fall K, Tchiakpe E, Ndiaye O, Ayouba A, et al. **Dried blood spots for HIV-1 drug resistance genotyping in decentralized settings in Senegal**. *J Med Virol* 2014; 86(1):45-51.

39. Hughes JP, Baeten JM, Lingappa JR, Magaret AS, Wald A, de Bruyn G, et al. **Determinants of per-coital-act HIV-1 infectivity among African HIV-1-serodiscordant couples**. *J Infect Dis* 2012; 205(3):358-365.

40. Weller S, Davis K. **Condom effectiveness in reducing heterosexual HIV transmission**. *Cochrane Database Syst Rev* 2001; (3):CD003255.

41. Baggaley RF, White RG, Boily MC. **HIV transmission risk through anal intercourse: systematic review, meta-analysis and implications for HIV prevention**. *Int J Epidemiol* 2010; 39(4):1048-1063.

42. Siegfried N, Muller M, Deeks JJ, Volmink J. **Male circumcision for prevention of heterosexual acquisition of HIV in men**. *Cochrane Database Syst Rev* 2009; (2):CD003362.
